# (+)-JQ-1 alleviates cardiac injury in myocardial infarction by inhibiting ferroptosis through the NAMPT/SIRT1 pathway

**DOI:** 10.1038/s41419-025-07880-x

**Published:** 2025-07-23

**Authors:** Mengxue Yang, Ting Wang, Jingrong Shao, Xinna Ran, Rui Xiao, Rui Zhao, Chunyan Wu, Ming Ji, Weiping Tian, Huabing Sun, Jiao Liu, Shengkai Zuo

**Affiliations:** 1 Department of Biopharmaceutics, Tianjin Key Laboratory of Technologies Enabling Development of Clinical Therapeutics and Diagnostics, Key Laboratory of Immune Microenvironment and Disease (Ministry of Education), The Province and Ministry Co-sponsored Collaborative Innovation Center for Medical Epigenetics, School of Pharmacy, Tianjin Medical University, Tianjin, China; 2 Research Center of Basic Medical Science, Tianjin Medical University, Tianjin, China; 3 Department of Chemical Biology, School of Pharmacy, Tianjin Medical University, Tianjin, China; 4 Department of Pharmacology, Tianjin Key Laboratory of Inflammatory Biology, School of Basic Medical Sciences, Tianjin Medical University, Tianjin, China

**Keywords:** Myocardial infarction, Diseases

## Abstract

Myocardial infarction (MI) remains one of the leading causes of mortality worldwide, and cardiomyocyte death plays a critical role in cardiac remodeling after MI. Ferroptosis is a recently identified form of iron-dependent programmed cell death that has been shown to be involved in the progression of various cardiovascular diseases, including MI. Bromodomain-containing protein 4 (BRD4) is an epigenetic reader and a key regulator of cell survival. In this study, we screened an epigenetic target library containing 773 small-molecule compounds and found that (+)-JQ-1(hereafter abbreviated as JQ-1), a BRD4-specific inhibitor, markedly attenuated ferroptosis induced by erastin (a ferroptosis inducer) in cardiomyocytes. Both prophylactic and therapeutic JQ-1 administration significantly improved cardiac remodeling and reduced cardiomyocyte ferroptosis in mice with MI. Mechanistically, JQ-1 protected cardiomyocytes from erastin-induced ferroptosis by downregulating the expression of nicotinate phosphoribosyltransferase (NAPRT) and upregulating the expression of nicotinamide phosphoribosyltransferase (NAMPT) and sirtuin1 (SIRT1). Inhibition of NAMPT or SIRT1 abrogated the protection conferred by JQ-1 in erastin-treated H9C2 cardiomyocytes. The combination of proteolysis-targeting chimeras (PROTACs) with JQ-1 (JQ-1-PROTAC) promoted BRD4 protein degradation and rescued erastin-induced ferroptosis in H9C2 cardiomyocytes, and prevented erastin-induced ferroptosis in human cardiomyocytes. Thus, JQ-1 can protect cardiomyocytes from ferroptosis through the NAMPT-SIRT1 pathway, and JQ-1-based therapy may serve as a novel promising strategy to improve cardiac remodeling after MI.

## Introduction

Cardiovascular disease continues to be the leading cause of disability and death in the world, accounting for approximately 18.6 million deaths each year [1, 2]. Myocardial infarction (MI) is the most common type of cardiovascular disease and the most severe clinical manifestation of coronary artery disease. The most common cause of MI is rupture of atherosclerotic plaques, which leads to occlusion of the coronary arteries of the heart and results in dramatic and irreversible loss of numerous cardiomyocytes in the infarcted area due to hypoxia and stress, subsequent inflammation and fibrotic scarring, and eventual progressive remodeling and heart failure [3]. Although percutaneous coronary interventional reperfusion immediately after MI can reduce the infarct area, it results in cardiomyocyte death owing to the production of reactive oxygen species (ROS) [4]. Much of the cardiomyocyte death during MI or reperfusion is mediated by programmed cell death signals, such as apoptosis, necroptosis, pyroptosis, autophagy, and the recently discovered ferroptosis [5–7]. Accumulating evidence indicates that pharmacological and genetic inhibition of cardiomyocyte death can diminish the infarct size and improve cardiac remodeling post-MI [8]. Since adult cardiomyocytes are terminally differentiated cells, identification of new interventions to reduce cardiomyocyte death is an important strategy for improving cardiac remodeling after MI.

Ferroptosis is a recently discovered iron-dependent programmed cell death that is characterized by iron overload, which leads to elevated lipid peroxidation, ROS accumulation, and mitochondrial abnormalities, and involved in the progression of ischemic cardiomyopathy [6, 8]. Lipid peroxidation and Glutathione Peroxidase 4 (GPX4) inactivation are hallmarks of ferroptosis. GPX4 expression is decreased in the pre- and metaphase of MI in a mouse model, and GPX4 inhibition leads to lipid peroxide accumulation and ferroptosis in cardiomyocytes [9]. Furthermore, nanosponge-based nanomedicines with iron chelation and efflux have been shown to significantly suppress cardiomyocyte ferroptosis and improve cardiac remodeling post-MI [10]. Nicotinamide phosphoribosyltransferase (NAMPT) and nicotinate phosphoribosyltransferase (NAPRT) are two master enzymes that catalyze the biosynthesis of nicotinamide adenine dinucleotide (NAD^+^), which is essential for energy homeostasis and ROS elimination by sirtuin 1 (SIRT1) [11, 12]. Enhancement of the NAMPT-SIRT1 axis by ferroptosis-associated circular RNA (FEACR) has been shown to inhibit hypoxia and reoxygenation-induced ferroptosis and alleviate myocardial ischemia and reperfusion injury [13]. Similarly, resveratrol, an activator of SIRT1, was shown to attenuate MI-induced cardiac dysfunction and fibrosis by reducing cardiomyocyte ferroptosis and increasing KAT5-dependent GPX4 expression in rats [14]. Therefore, the identification of new drugs or targets that can inhibit ferroptosis in cardiomyocytes is of great importance for the treatment of MI.

Epigenetics is the process of regulating the function/expression/activity of alternative genes through chemical modifications that affect gene expression without altering the sequence of DNA. Epigenetics is thought to be the primary regulatory strategy for cellular responses to environmental changes [15]. Increasing evidence indicates that epigenetics is deeply involved in the regulation of ferroptosis [16]. As a member of the bromodomain and extra-terminal (BET) family, bromodomain-containing protein 4 (BRD4) recognizes acetylation sites in promoter regions and recruits transcription factors to regulate target gene expression. Although inhibition of BRD4 suppresses cardiac hypertrophy and pathologic cardiac remodeling [17, 18], cardiomyocyte-specific deletion of BRD4 in mice triggers progressive deterioration of cardiac contractile function and eventually lead to dilated cardiomyopathy [19]. Notably, (+)-JQ-1 (hereafter abbreviated as JQ-1), a small-molecule inhibitor of BRD4, has been reported to suppress cardiomyocyte apoptosis and improve cardiac remodeling in ischemic heart injury [18, 20–22], indicating that JQ-1 may be a new therapeutic agent for ischemic heart disease. However, the effects of JQ-1 on cardiomyocyte ferroptosis and its exact role in MI remain unknown.

Here, we identified JQ-1 from an epigenetic compound library in an erastin-induced cell ferroptosis model in a completely unbiased manner. JQ-1 reduced erastin-induced ferroptosis and ROS production in cardiomyocytes. Whether administered before or after MI, JQ-1 significantly improved cardiac remodeling by reducing ferroptosis of cardiomyocytes. JQ-1 promoted SIRT1 expression in infarcted hearts by modifying the balance between NAMPT and NAPRT, and inhibition of SIRT1 abrogated the protection conferred by JQ-1 in erastin-treated cardiomyocytes. Furthermore, the combination of proteolysis-targeting chimeras (PROTACs) with JQ-1 (JQ-1-PROTAC) rescued erastin-induced ferroptosis in rat and human cardiomyocytes by inducing BRD4 degradation. The identification of JQ-1 as an anti-ferroptosis molecule in cardiomyocytes paves the way for the development of novel, promising small-molecule compounds to improve cardiac remodeling after MI.

## Materials and methods

### Animals

All mice were maintained on a C57/BL6 background, used in accordance with the guidelines of the National Institutes of Health (NIH) Guide for the Care and Use of Laboratory Animals. All mice were housed in temperature-controlled rooms under a 12-hour light/12-hour dark cycle and provided with an adequate amount of chow and water. The sample size is described in the corresponding figure legend. Subsequently, animals were randomized into different groups. No blinding was performed. No animals were excluded from the analysis.

As described previously [23], permanent ligation of the left anterior descending branch of the coronary artery (LAD) in 8-week old male mice to construct an MI model. Briefly, the experimental mice were anesthetized with 1.0–1.5% isoflurane (R510; RWD Life Science, Shenzhen, China) throughout surgery. MI models were established by ligating the LAD with 8–0 silk sutures 2–3 mm below the left auricle and puncturing without ligation in the sham-operated group.

Throughout all mouse experiments, vehicle or JQ-1 (50 mg/kg) was administered daily via intraperitoneal injection. To prepare the JQ-1 solution, pure solvents were added to JQ-1 (dissolved in 5% dimethyl sulfoxide [DMSO]) in the following order: 40% PEG300 (IP9020; Solarbio, Beijing, China), 5% Tween80 (IT9000; Solarbio, China), and 50% double-distilled water (ddH_2_O). The vehicle consisted of 5% DMSO, 5% Tween80, 40% PEG300, and 50% ddH_2_O.

### Reagents

Erastin (#S7242), JQ-1(#S7110), and EX527(#S1541) were purchased from Selleck Chemicals (Texas, USA). JQ-1-PROTAC was grant from Huabing Sun’s Lab (Tianjin Medical University). FK866 (#T2644) and ML385 (#T4360) were obtained from TargetMol (Massachusetts, USA). Ferrostatin-1 (#HY-100579) was purchased from MedChemExpress Company (Shanghai, China).

### Cell culture and treatment

H9C2 rat cardiomyocytes and AC16 human cardiomyocytes were obtained from Shanghai Zhong Qiao Xin Zhou Biotechnology Co., Ltd. (Shanghai, China) and were cultured with Dulbecco’s modified Eagle medium (DMEM; C11995500BT; Gibco, California, USA) containing 10% (vol/vol) fetal bovine serum (MN012103, Mengma, Tianjin, China) and 50 μg/mL penicillin-streptomycin (FG101-01; TransGen Biotech, Beijing, China). Cells were cultured at 37 °C in a humidified cell culture incubator wtih 5% CO_2_. All cells used in this study were free of mycoplasma contamination and were validated using the short tandem repeat (STR) method.

NRVMs were isolated from 1-day-old neonatal SpragueDawley rats [24]. Briefly, neonatal rat hearts were separated and dissociated using 0.25% trypsin (15090046; Gibco, USA). Rat heart tissue was digested in 0.1% collagenase typeII (LS004176; Worthington, North Carolina, USA) at 37 °C. NRVMs were collected and seeded on collagen in DMEM medium containing 10% (vol/vol) fetal bovine serum and 50 μg/mL penicillin-streptomycin. After 24 h, the medium was replaced with DMEM, which was used for follow-up experiments. NRVMs were cultured at 37 °C in a humidified cell culture incubator with 5% CO_2_.

### Cell viability assay

Cell viability was evaluated using the Cell Counting Kit-8 (CCK-8) (C6005; NCM Biotech, Suzhou, China) in accordance with the manufacturer’s instructions. Cells were seeded in 96-well plates (6000 cells per well) and treated with erastin (2.5 μM) for 24 h to induce cardiomyocyte ferroptosis. The compounds utilized in this study included JQ-1 (1 μM), JQ-1-PROTAC (0.5 μM), EX527 (10 μM), and FK866 (10 μM). Ferrostatin-1 (Fer-1, 2 μM), a conventional ferroptosis inhibitor, was used as the positive control to validate the cardiomyocyte ferroptosis model. All inhibitors were administered to cells 2 h before erastin stimulation. After drug treatment, 100 μL of 10% CCK-8 solution or an equivalent volume of cell culture medium was added to the corresponding wells as required, with the cell-free group serving as the blank control. The absorbance value of each well was measured at 450 nm using a microplate reader (PerkinElmer, Massachusetts, USA). The proportion of surviving cells in each group was subsequently normalized to the proportion of surviving cells in the control wells.

### High-throughput screening

H9C2 cardiomyocytes were seeded in 96-well plates at a density of about 70%-80% and cultured overnight. Subsequently, a total of 773 compounds, belonging to the epigenetic compound library (L1200; TargetMol, USA), were added to the cell culture medium at a final concentration of 5 μM using the Explorer G3 Automated Drug Screening System (PerkinElmer, USA). Next, erastin was added to the cells and continued incubation for 24 h. The viability of the cells is then assessed using the CCK-8 assay.

### Bright-field assay

H9C2 cardiomyocytes were seeded in 6-well plates at a density of about 70–80% and treated with JQ-1 for 24 h. The cells were then examined under the bright field of a trinocular inverted fluorescence microscope (WYS-41XDY; VIYEE, Tianjin, China).

### Calcein AM and propidium iodide (PI) staining

H9C2 cardiomyocytes were seeded in 12-well plates at a density of about 70–80% and subsequently treated with the specified compounds 24 h later. The cells were washed twice with phosphate-buffered saline (PBS), after which a certain volume of Calcein AM/PI assay solution (C2015M; Beyotime Biotech, Shanghai, China) was added. The samples were then incubated at 37 °C for 30 min away from light. Subsequently, the samples were examined for fluorescence under a trinocular inverted fluorescence microscope (Calcein AM, green fluorescence; excitation/emission, 494/517 nm; PI, red fluorescence; excitation/emission, 535/617 nm). The reaction mixture was shielded from light throughout the process.

### ROS detection

ROS Assay Kit (S0033S; Beyotime Biotech, China) are utilized in accordance with the instructions provided by the manufacturer for the measurement of intracellular ROS levels. Briefly, the cardiomyocytes in the 12-well plates were treated with indicated drugs for 24 h, washed twice with PBS, and diluted fluorescent probes (1:1000) were added, and then the cells were incubated for 30 min at 37 °C. After that, the cells were washed twice with serum-free DMEM and observed under a trinocular inverted fluorescence microscope. The intensity of cellular fluorescence was quantified using ImageJ software (version 6.0; Media Cybernetics, Maryland, USA).

Intracellular ROS levels were quantified using flow cytometry. First, anchorage-dependent H9C2 cardiomyocytes were suspended in 0.05% trypsin (25200072; Gibco, USA). Then, the suspended cells were subjected to centrifugation at 1000 rpm for 5 min at 4 °C. Following this, the cleaned cells were suspended in diluted DCFH-DA; and the cells were then incubated for 30 min at 37 °C. It is essential to ensure that the probe maintains adequate contact with the cells by mixing the solution upside down at regular intervals, approximately every 3–5 min. Subsequently, the cells were washed 3 times with a serum-free cell culture solution in order to completely remove the DCFH-DA probe that had not enter the cells. The ROS levels in each sample were determined by flow cytometry (BD Biosciences, New Jersey, USA) and analyzed by FlowJo software.

### C11-BODIPY assay

The C11-BODIPY assay was conducted in accordance with the instructions provided in the lipid peroxidation kit (L267; DOJINDO, Kyushu, Japan). H9C2 cardiomyocytes in 12-well plates were subjected to a 24-h challenge with the specified drugs. Following this, the cells were washed twice with Hanks’ balanced salt solution (HBSS; C0219; Beyotime Biotech, China); and the prepared working solution was then added and the cells were incubated in an incubator for 30 min at 37 °C and 5% CO_2_. After Hoechst stain in HBSS was used to stain the nucleus for 10 min, the cells were then washed 2 times with HBSS and examined under a trinocular inverted fluorescence microscope. The fluorescent probe fluoresces red normally, but the fluorescence changes from red to green as lipid peroxidation occurs. The fluorescence intensities of the two colors can be used to detect the formation of lipid peroxidation products with high sensitivity. Cellular green fluorescence intensities were quantified using ImageJ software 6.0.

### Western blotting

Radioimmunoprecipitation assay (RIPA) lysis buffer (P0013C; Beyotime Biotech, China) containing a protease inhibitor cocktail (C0001; TargetMol, USA) was used to lyse cardiomyocytes or cardiac tissues for 30 min to obtain proteins on ice. The above lysate was subjected to centrifugation at 12,000 × *g* for 15 min at 4 °C to obtain the supernatant as a protein solution, and the total protein concentration was determined using the Pierce™ BCA Protein Assay Kit (23225; Thermo Fisher Scientific, Massachusetts, USA). The proteins were denatured at the same concentration for 10 min, separated using 10% sodium dodecyl sulfate-polyacrylamide gel electrophoresis (SDS-PAGE), and then transferred to polyvinylidene difluoride (PVDF) membranes (IPVH00010; Merck Millipore, New Jersey, USA). Then, the membrane containing the protein was blocked with 5% non-fat milk for 1–2 h at room temperature (RT), after which it was incubated with specific primary antibodies overnight at 4 °C. The following primary antibodies were used in this study: GPX4 (A11243, 1:1000; ABclonal Technology, Wuhan, China), BRD4 (A18840, 1:1000; ABclonal Technology, China), Nrf2 (A3577, 1:1000, ABclonal Technology, China), p-Nrf2 (Ap1133, 1:1000, ABclonal Technology, China), SLC7A11(A2413, 1:1000, ABclonal Technology, China), FPN (26601-1-AP, 1:1000, proteintech, Chicago, USA), and GAPDH (AC002, 1:10000; ABclonal Technology, China). After washing in TBST for 3 times, the protein-containing membrane bound to the primary antibody was incubated with the secondary antibody (7074 V, 1:2000; Cell Signaling Technology, Massachusetts, USA) for 1–2 h at RT. The blotted bands were identified through the utilization of an enhanced chemiluminescence assay (34580; Thermo Fisher Scientific, USA) and quantified using ImageJ software. GAPDH was employed as the control for data normalization.

### Immunofluorescence

NRVMs were first gently rinsed three times with PBS. Next, they were fixed in 4% paraformaldehyde (PFA) at RT for 30 min. After fixation, cells were permeabilized with 0.1% Triton X-100 for 15 min, followed by blocking with 3% bovine serum albumin (BSA) for 60 min at RT. Following washing with PBS, the primary antibody, cTnT (MA5-12960, 1:100 dilution, Invitrogen, California, USA), was added, and cells were incubated at 4 °C overnight. The next day, cells were washed three times with PBS, and the secondary antibody (Alexa Fluor 488-conjugated goat anti-mouse IgG, A-11029, 1:2000 dilution, Invitrogen, USA) was added. Samples were incubated for 60 min at RT in the dark to prevent photobleaching. After final washes, slides were mounted with a DAPI-containing mounting agent to counterstain nuclei and visualized under a fluorescence microscope for image acquisition.

### Quantitative real-time polymerase chain reaction (RT-PCR)

The TRIzol reagent (15596018; Invitrogen, USA) is employed for the purpose of extracting total RNA from cardiac tissue or cardiomyocytes. cDNA was obtained through cDNA Synthesis using the SuperMix (11141es60; Yeasen Biotech, Shanghai, China). Reverse transcription quantitative polymerase chain reaction (RT-PCR) was conducted using SYBR Green Mix (11184es08; Yeasen Biotech, China). Primers were obtained from GeneWiz Biotechnology (Shanghai, China). The specific mRNA levels were calculated using the standard comparative CT method, with the results being normalized to the levels of *Gapdh*. The primer sequences for these genes are listed in Supplementary Table 2.

### Chromatin immunoprecipitation (ChIP)-PCR

ChIP-PCR was performed as previously described [25]. Briefly, H9C2 cells were cross-linked with 1% formaldehyde for 10 min, and the reaction was quenched with 125 mM glycine. Genomic DNA was then sheared into fragments (200–500 bp) using a Bioruptor Sonicator (Diagenode) according to the manufacturer’s protocol. For immunoprecipitation, cell lysates were incubated overnight at 4 °C with 4 μg of anti-BRD4 antibody (67374-2-Ig, Proteintech) or control IgG (12–371, Millipore). Following washes and elution, protein-DNA cross-links were reversed by heating at 65 °C for 6 h. DNA fragments enriched by ChIP were purified and detected via RT-PCR using *NAPRT* specific primers (Supplementary Table 3).

### Determination of GSH levels

GSH levels in cardiomyocytes or cardiac tissues were quantified using a GSH and GSSG Assay Kit (S0053; Beyotime Biotech, China), in accordance with the manufacturer’s instructions. Briefly, H9C2 cardiomyocytes or cardiac tissues after grinding with liquid nitrogen were collected in a 1.5 mL tube, centrifuged, and the supernatant was subsequently removed. Next, three volumes of the cell pellet protein removal reagent M solution was added, and the mixture was thoroughly vortexed. The samples were subjected to rapid freeze-thaw cycles on two occasions, alternating between liquid nitrogen and 37 °C water bath. Following a 5 min period of cooling on ice, the samples were subjected to centrifugation at 10,000 × *g* for 10 min at 4 °C, and the resulting supernatant was obtained for the purpose of measuring the total GSH content. A proportion of the aforementioned samples should be combined with the GSH clearance auxiliary solution and GSH clearance reagent working solution in accordance with a 5:1 ratio, vortex mixing without delay. Following a 60-min reaction at 25 °C, the standards and samples were introduced to the 96-well plate in a sequential manner, then 150 μL of total glutathione detection solution was added, followed by 50 μL of 0.5 mg/mL NADPH solution. Following a 25-min incubation period, and the absorbance value was subsequently measured using a microplate reader at 412 nm. Standard curves were made based on the different absorbances measured by different concentration standards. The sample is compared to the standard curve to calculate the total GSH and GSSG contents.

### Determination of MDA levels

MDA levels in H9C2 cardiomyocytes or cardiac tissues were quantified using a Lipid Peroxidation MDA Assay Kit (S0131S; Beyotime Biotech, China). Briefly, H9C2 cardiomyocytes or cardiac tissues were lysed in western lysis buffer (P0013C; Beyotime Biotech, China). The supernatant was collected by centrifugation at 10,000–12,000 × *g* for 10 min at RT, and measure protein concentration using the BCA protein assay (23225; Thermo Fisher Scientific, USA), and 100 μL of the sample was added for the assay, followed by 200 μL of the MDA assay solution. After vortexing to mix, heat the mixture 15 min at a 100 °C metal bath. Thereafter, the solution be cooled to RT in a water bath, and then subjected to centrifugation at 1000 × *g* for 10 min at RT. Next, 200 μL of the resulting supernatant was added to a 96-well plate, and the absorbance value was subsequently measured with a microplate reader at 532 nm. For H9C2 cardiomyocytes or cardiac tissues, the level of MDA in the initial sample can be expressed as the protein content per unit or cardiac tissue weight after the MDA content in the sample solution has been calculated.

### Determination of NAD^+^ levels

NAD^+^ levels were quantified using a NAD^+^/NADH Assay Kit with WST-8 (S0175; Beyotime Biotech, China) according to the instructions provided.

### Echocardiography

At 7, 14, and 28 days after MI, the mice were anaesthetized with 1.0–1.5% isoflurane (R510; RWD Life Science, China), and cardiac function was assessed by obtaining M-mode echocardiography images of the left ventricle using an echocardiography machine (Visual Sonics, Toronto, Canada). Heart rate, the left ventricular ejection fraction, and left ventricular fractional shortening were calculated using the Visual Sonics Vevo 2100 ultrasound imaging system, as described previously [26]. At 28 days after MI, the mice were weighed and euthanized using an overdose of chloral hydrate (C104202; Aladdin Biotech, Shanghai, China), and the cardiac tissues were harvested for further analysis.

### Histological analysis

Mouse heart tissue samples were washed with PBS to remove blood, fixed in 4% paraformaldehyde at 4 °C for more than 48 h, and sectioned by paraffin embedding. Tissue sections of 6 μm thickness were obtained on a HistoCore Arcadia paraffin-embedding machine (Leica, Wetzlar, Germany). Next, sections staining was carried out using a hematoxylin and eosin staining kit (G1120; Servicebio, China) and Masson trichrome staining kit (G1340; Servicebio, China) according to the manufacturer’s protocol. Stained sections were observed and photographed using on a Leica microscope (Leica, Germany). The percentages of fibrotic area and infarct areas were calculated using ImageJ software 6.0, as described in previous studies [27].

### Transmission electron microscopy

Small pieces of approximately 1 mm [3] were cut from the infarcted border zone of the left ventricle of the harvested mouse hearts. Samples were then fixed in an electron microscope fixation solution (G1102; Servicebio, China) for 24 h and post-fixed with 1% osmium tetroxide (201030; Sigma Aldrich, Missouri, USA) in 0.1 M sodium cacodylate buffer (C0250; Sigma Aldrich, USA) for one hour. The next stage of the process involved dehydrating the pieces, embedding them in resin, and cutting them into sections with 80-nm-thick. All images of cardiac mitochondrial were obtained using a transmission electron microscope (HT-7800; Hitachi, Tokyo, Japan) at ×3000 and ×10,000 magnification. Mitochondrial morphology scoring was performed in accordance with the rules described previously [28].

### Statistical analyses

Data were expressed as the mean ± standard error of the mean (SEM). All data were subjected to a Shapiro-Wilk normality test, which confirmed that they were normally distributed. Two-group data were compared using a two-tailed Student’s *t* test. In the case of multiple-group comparisons and comparisons of two-factor multiple group comparisons, one-way and two-way ANOVA were employed, respectively. The statistical analysis was conducted using GraphPad Prism 8 (GraphPad Software, Inc., San Diego, USA), and *P* < 0.05 was considered statistically significant.

## Results

### Identification of the rescue effects of JQ-1 on erastin-induced cardiomyocyte ferroptosis

**Fig. 1:**
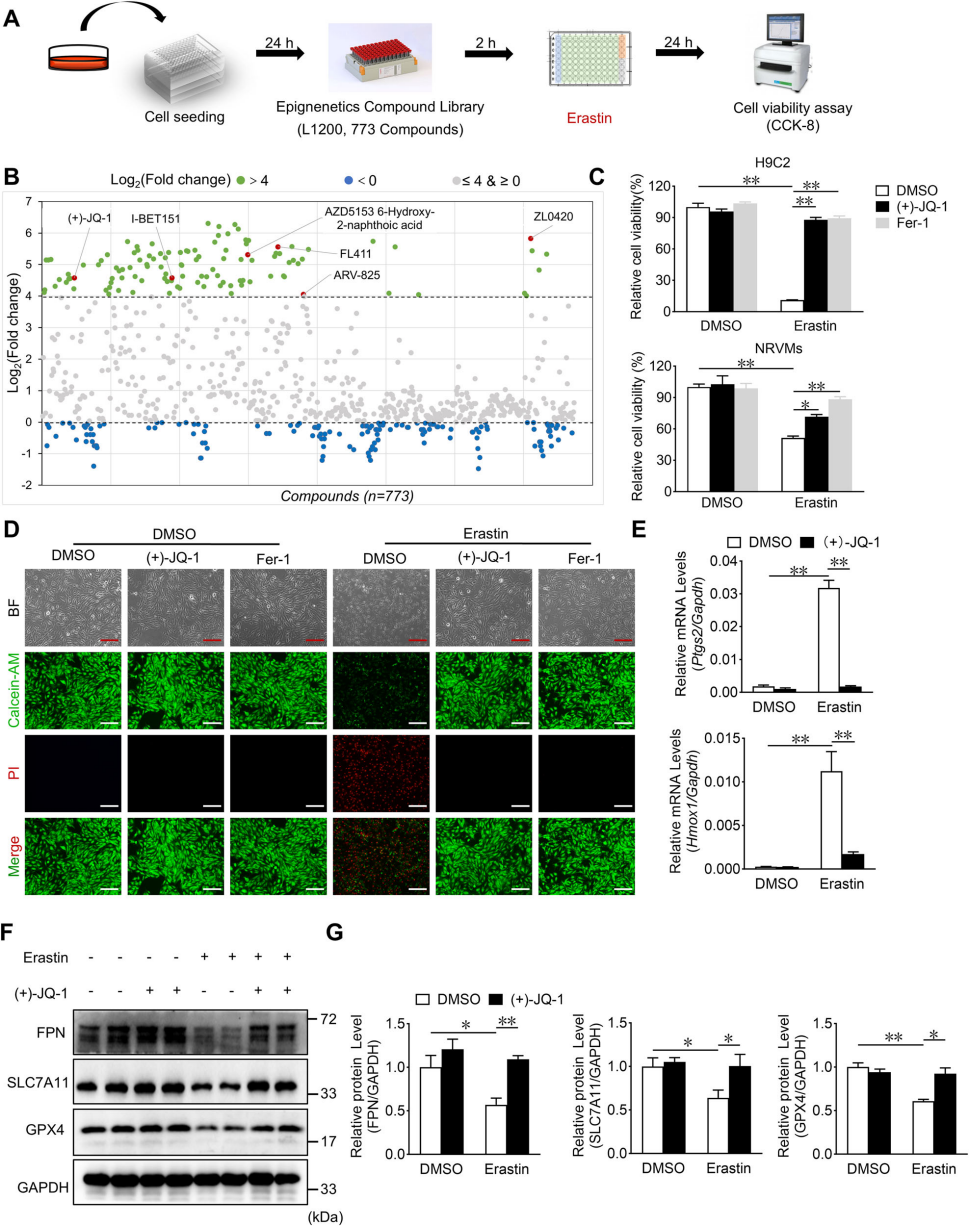
Identification of JQ-1 as the candidate compound for the inhibition of cardiomyocyte ferroptosis. **A** Screening strategy for small molecular from epigenetic compounds library in H9C2 cardiomyocytes. **B** Scatter plot of the effect of small molecule compounds in the library on erastin-induced cellular viability in H9C2 cardiomyocytes. Green dots [Log_2_(Fold change) > 4] indicate anti-ferroptosis candidate compounds, blue dots [Log_2_(Fold change) < 0] indicate pro-ferroptosis candidate compounds, gray dots [0≦Log_2_(Fold change) ≦4] indicate compounds that have no effect on ferroptosis. **C** Effect of JQ-1 (1 μM) on the cell viability in H9C2 cardiomyocytes (up) and neonatal rat ventricular myocytes (NRVMs, down) treated with erastin (2.5 μM). The positive control was represented by the use of Fer-1 (2 μM) (n = 5–6). **D** The representative images of bright field (BF), Calcein-AM, and propidium iodide (PI) staining in H9C2 cardiomyocytes treated with JQ-1 under the erastin challenge. Green indicates the fluorescence of Calcein-AM, red indicates the fluorescence of PI. Scale bars, 100 µm, (n = 6). **E** The relative mRNA levels of *Ptgs2* and *Homx1* in JQ-1-treated H9C2 cardiomyocytes under the erastin challenge (n = 4). **F** Western blot analysis was conducted to assess the expression of FPN, SLC7A11, and GPX4 in JQ-1-treated H9C2 cardiomyocytes under the erastin challenge. **G** The relative protein levels of FPN, SLC7A11, and GPX4 were quantified in (**F**) (n = 4). The data are presented as mean ± SEM. The statistical significance of the data was evaluated using a two-way ANOVA followed by Tukey’s test for multiple comparisons (**C**, **E**, and **G**). *P < 0.05; **P < 0.01.

To identify small-molecule compounds that can rescue ferroptosis in cardiomyocytes, we utilized an erastin-induced cell ferroptosis model to screen an epigenetics compound library in H9C2 cardiomyocytes (Fig. 1A). The epigenetics compound library had 773 compounds, including a majority of currently available target compounds of epigenetic regulatory enzymes. We ranked the viability of H9C2 cardiomyocytes treated with these compounds based on CCK-8 assay results. A total of 115 compounds significantly rescued the erastin-induced decline in cardiomyocyte viability, of which six targeted BRD4 (Fig. 1B and Supplementary Table 1). BRD4 is abundantly expressed in the heart, and both the mRNA and protein levels of BRD4 are elevated in cardiomyocytes after MI [21, 22]. In addition, because JQ-1 is currently the most studied BRD4 inhibitor with great application potential, we chose JQ-1 as a candidate small-molecule compound for further studies.

To validate the role of JQ-1 in ferroptosis, we selected ferrostatin-1 (Fer-1, a classic ferroptosis inhibitor) as the positive control. CCK-8 assay results showed that JQ-1, like Fer-1, markedly inhibited erastin-induced ferroptosis in H9C2 cells (Fig. 1C). JQ-1 protected against erastin-induced cell death in H9C2 cells in a dose-dependent manner (Supplementary Fig. 1). We isolated and identified neonatal rat ventricular myocytes (NRVMs) and found that JQ-1 also had a protective effect on NRVMs (Supplementary Fig. 2A and Fig. 1C). We then used Calcein-AM to detect the relative levels of intracellular free iron, and erastin treatment was found to trigger a markedly decrease in calcein fluorescence intensity (indicating increased free iron levels), whereas JQ-1 or Fer-1 markedly increased the fluorescence intensity of calcein after erastin treatment in H9C2 cells (Fig. 1D). Bright-field micrographs and propidium iodide (PI) staining showed that JQ-1 or Fer-1 treatment completely rescued erastin-induced ferroptosis in cardiomyocytes (Fig. 1D). In addition, JQ-1 restored erastin-decreased calcein fluorescence and inhibited erastin-increased PI fluorescence in NRVMs (Supplementary Fig. 2B). Importantly, JQ-1 treatment blocked the erastin-induced increase in the expression of prostaglandin-endoperoxide synthase 2 (*Ptgs2*) and heme oxygenase-1 (*Hmox1*), two pro-ferroptosis marker genes (Fig. 1E). Consistent with this finding, western blot analysis showed that JQ-1 treatment blocked the erastin-induced reduction in of ferroptosis-inhibiting proteins, including solute carrier family 7 member 11 (SLC7A11), ferroportin (FPN) and GPX4 levels in cardiomyocytes (Fig. 1F, G). These results suggest that JQ-1 is a small-molecule compound that effectively inhibits erastin-induced ferroptosis in cardiomyocytes.

### JQ-1 ameliorates erastin-induced ROS production and accumulation of lipid peroxidation products in cardiomyocytes

**Fig. 2:**
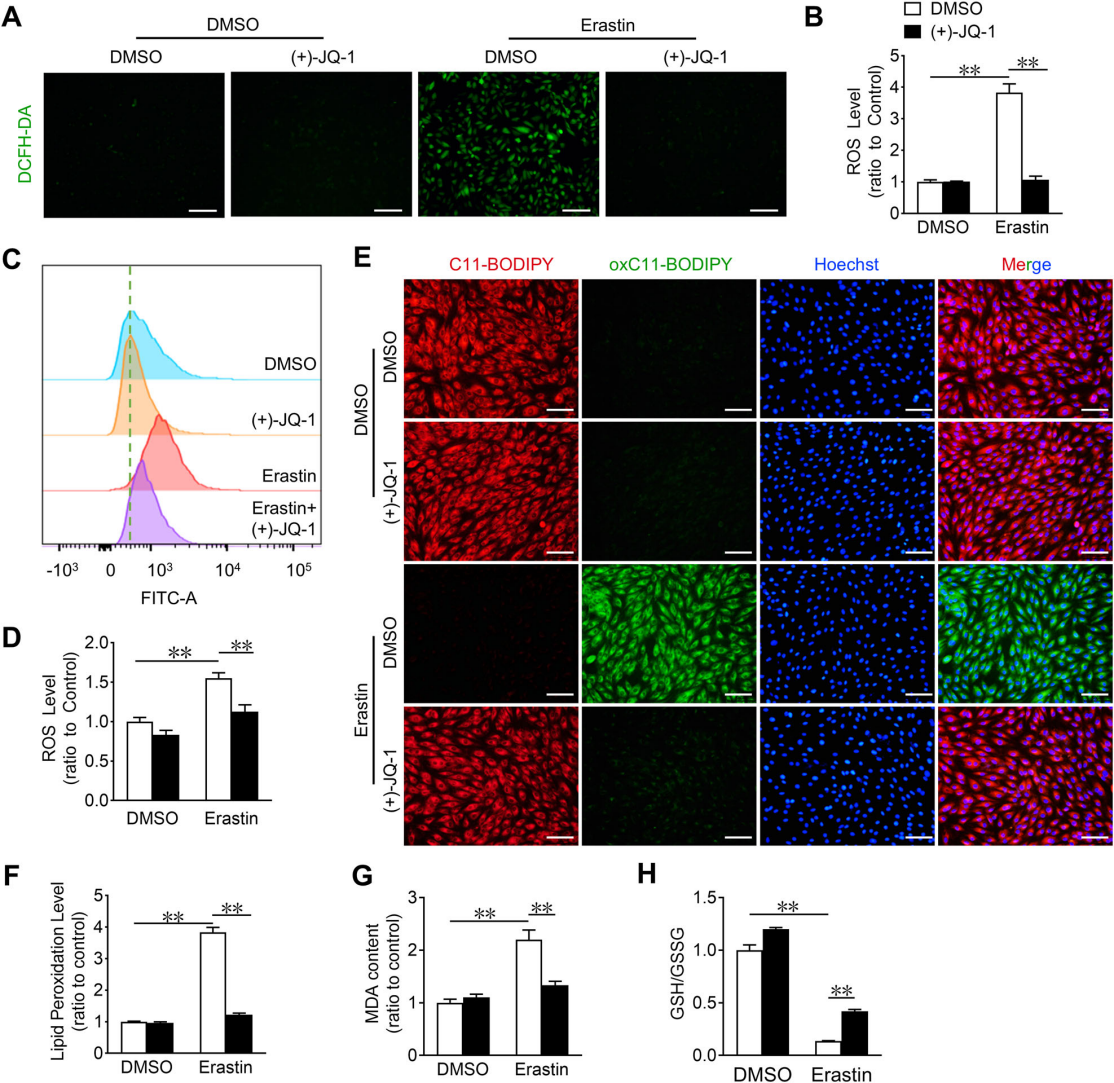
JQ-1 reduces ROS and lipid peroxidation in H9C2 cardiomyocytes after erastin treatment. **A** Representative DCFH-DA fluorescence (ROS probe) images in H9C2 cardiomyocytes treated with JQ-1 under the erastin challenge. Scale bars, 100 µm. **B** Quantitative analysis of ROS levels in H9C2 cardiomyocytes (n = 3). **C** Representative flow cytometric plots of H9C2 cardiomyocytes treated with JQ-1 under the erastin challenge. **D** Quantitative analysis of intracellular ROS levels in H9C2 cardiomyocytes (n = 3). **E** Representative lipid peroxidation staining images in H9C2 cardiomyocytes treated with JQ-1 under the erastin challenge. Red: unoxidized C11-BODIPY; Green: oxidized C11-BODIPY; Blue: Hoechst-stained nucleus. Scale bars, 50 μm. **F** Quantitative analysis of lipid peroxidation levels in (**E**) (n = 3). Quantitative analysis of MDA levels (**G**) and GSH/GSSG levels (**H**) in H9C2 cardiomyocytes treated with JQ-1 under the erastin challenge (n = 3). The data are presented as mean ± SEM. The statistical significance of the data was evaluated using a two-way ANOVA followed by Tukey’s test for multiple comparisons (**B**, **D**, **F**–**H**). **P < 0.01.

ROS production and accumulation of lipid peroxidation products are essential for ferroptosis in cells [29]. Dichlorodihydrofluorescein diacetate (DCFH-DA) staining showed that JQ-1 markedly suppressed erastin-induced ROS production in H9C2 cells and in NRVMs (Fig. 2A–D and Supplementary Fig. 2C, D). We then used a C11-BODIPY fluorescent probe to examine the effect of JQ-1 on cellular lipid peroxidation. JQ-1 treatment almost completely inhibited erastin-induced lipid peroxidation and fluorescence accumulation in H9C2 cells (Fig. 2E, F). Correspondingly, JQ-1 also eliminated the erastin-induced generation of lipid peroxidation-derived malondialdehyde (MDA) in erastin-treated H9C2 cells (Fig. 2G). Glutathione (GSH) to glutathione disulfide (GSSG) ratio is a reliable estimate of the cellular redox status [30]. JQ-1 consistently mitigated the erastin-induced decrease of the GSH/GSSG ratio in H9C2 cells (Fig. 2H). Thus, JQ-1 ameliorated erastin-induced ROS production and the accumulation of lipid peroxidation products in cardiomyocytes.

### JQ-1 administration alleviates cardiac injury after MI by reducing MI-induced ferroptosis in mice

**Fig. 3:**
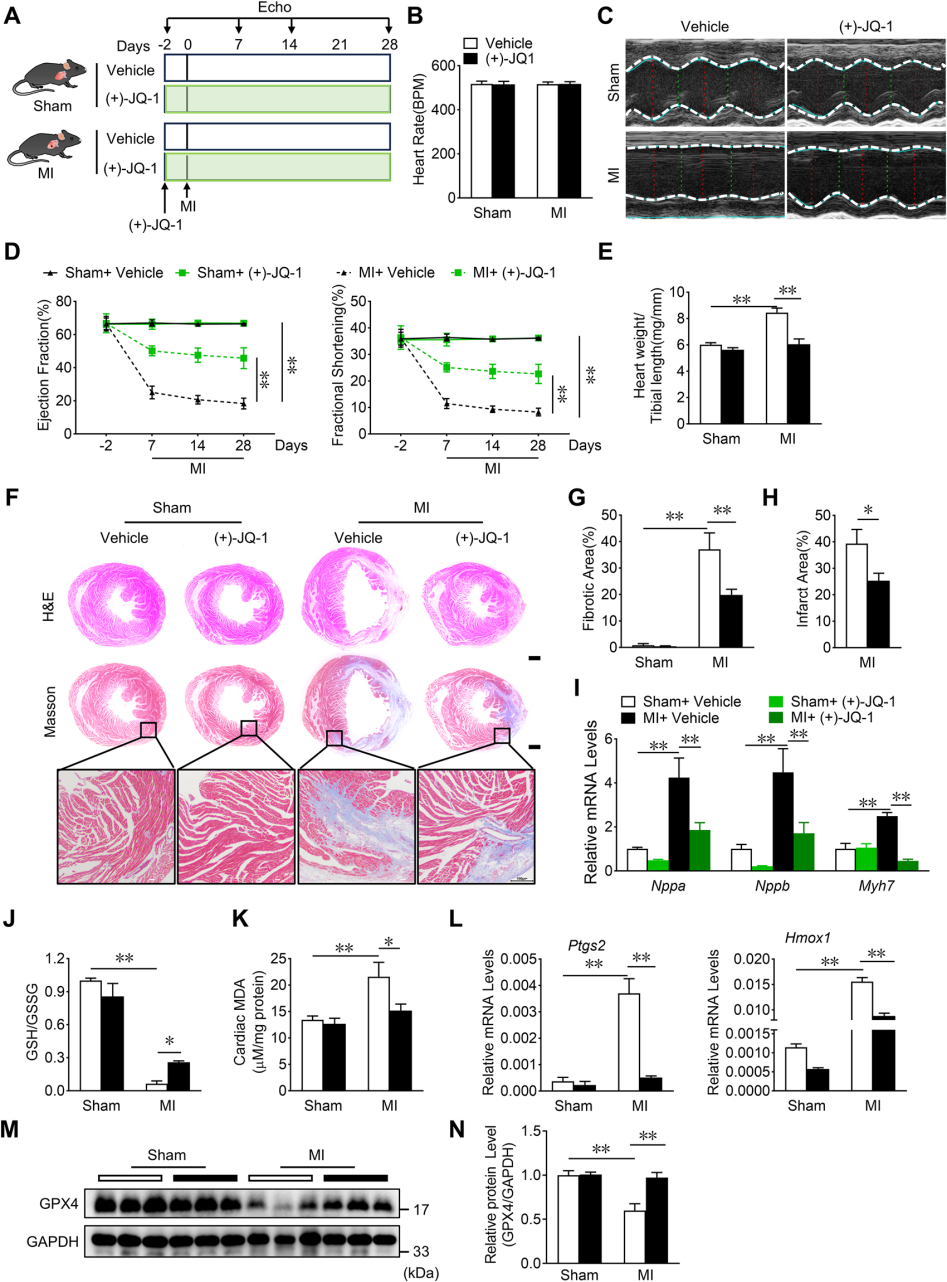
JQ-1 administration alleviates MI-induced cardiac injury in mice. **A** Experimental protocol showing JQ-1 administration strategy in mice with MI. JQ-1 (50 mg/kg) or equivalent vehicle was injected intraperitoneally 2 days before MI. **B** Heart rate of JQ-1-administrated mice after 28 days of MI (n = 6). **C** Representative M-mode echocardiography images in mice 28 days post MI treated with JQ-1. **D** Effect of JQ-1 administration on LVEF and LVFS at indicated time points (n = 6). **E** Effect of JQ-1 administration on heart weight to tibial length ratios 28 days after MI (n = 6). **F** Representative H&E and Masson’s trichrome stained heart sections from mice 28 days post MI treated with JQ-1. Scale bars, 1 mm. Quantification of the fibrosis area (**G**) and infarct area (**H**) of the myocardium from mice 28 days post MI with JQ-1 administration (n = 6). **I** Effect of JQ-1 on *Nppa*, *Nppb*, and *Myh7* mRNA expression in the cardiac tissue from mice 1 day after MI (n = 6). Effect of JQ-1 on cardiac GSH/GSSG ratios (**J**) and MDA levels (**K**) from mice 1 day after MI (n = 6). **L** Analysis of *Ptgs2* and *Hmox1* mRNA levels in the cardiac tissue from mice 1 day post-MI, (n = 3). Representative immunoblot images (**M**) and quantitative analysis (**N**) of the relative GPX4 protein levels in the cardiac tissue from mice 3 days post-MI (n = 3). The data are presented as mean ± SEM. The statistical significance of the data was evaluated using a Student’s two-tailed t test (**H**), and two-way ANOVA followed by Tukey’s test for multiple comparisons (**B**, **D**, **E**, **G**, **I**, **J**, **K**, **L**, and **N**). *P < 0.05; **P < 0.01.

Next, we used a murine model of permanent MI to determine the role of JQ-1 in vivo. JQ-1 was administered by intraperitoneal injection 2 days prior to MI and once daily thereafter until the end of the experiment. The same dose of the vehicle was administered to mice in the control group (Fig. 3A). JQ-1 treatment had no obvious effects on heart rate, cardiac function, heart weight to tibia length ratio, and myocardial fibrosis in sham-operated mice (Fig. 3B–G). In the MI group, JQ-1 remarkably attenuated cardiac dysfunction, as shown by the long-axis B-mode echocardiography images (Fig. 3C). We observed significant improvements in the left ventricular ejection fraction (LVEF) and left ventricular fraction shortening (LVFS) in the JQ-1 treatment group from 7 to 14 days and up to 28 days after MI (Fig. 3D). In addition, the heart rate of mice in the JQ-1 treatment group remained unchanged compared to the vehicle group (Fig. 3B), but JQ-1 treatment improved cardiac remodeling, as evidenced by lower heart weight/tibia length ratios, smaller infarct area, and less interstitial fibrosis 28 days after MI (Fig. 3E–H). Consistent with these findings, the mRNA levels of cardiac stress genes, including atrial natriuretic peptide (*Nppa*), brain natriuretic peptide (*Nppb*), and myosin heavy chain 7 (*Myh7*) was dramatically downregulated in JQ-1-treated mice after MI (Fig. 3I).

Mitochondria play important roles in cardiac remodeling and cardiomyocyte ferroptosis after MI, and mitochondrial function and morphology are significantly altered during cardiomyocyte ferroptosis [7, 31, 32]. Transmission electron microscopy images and quantification analysis indicated that MI led to changes in mitochondrial morphology and reduced spinal and cardiomyocyte mitochondria scores, which were rescued by JQ-1 treatment in MI hearts (Supplementary Fig. 3A, B). JQ-1 treatment increased the GSH/GSSG ratio and decreased the MDA levels in infarcted hearts (Fig. 3J, K). The expression of *Ptgs2* and *Hmox1* were markedly lower in JQ-1-treated hearts than in vehicle-treated hearts after MI (Fig. 3L). Moreover, JQ-1 treatment increased the level of the anti-ferroptosis protein GPX4 in cardiac tissue in comparison with that in the vehicle group after MI (Fig. 3M, N). These results suggest that treatment with JQ-1 initiated two days before MI attenuated the MI-induced cardiac injury and dysfunction by reducing cardiomyocyte ferroptosis.

### Therapeutic JQ-1 administration improves MI-induced cardiac remodeling in mice

**Fig. 4:**
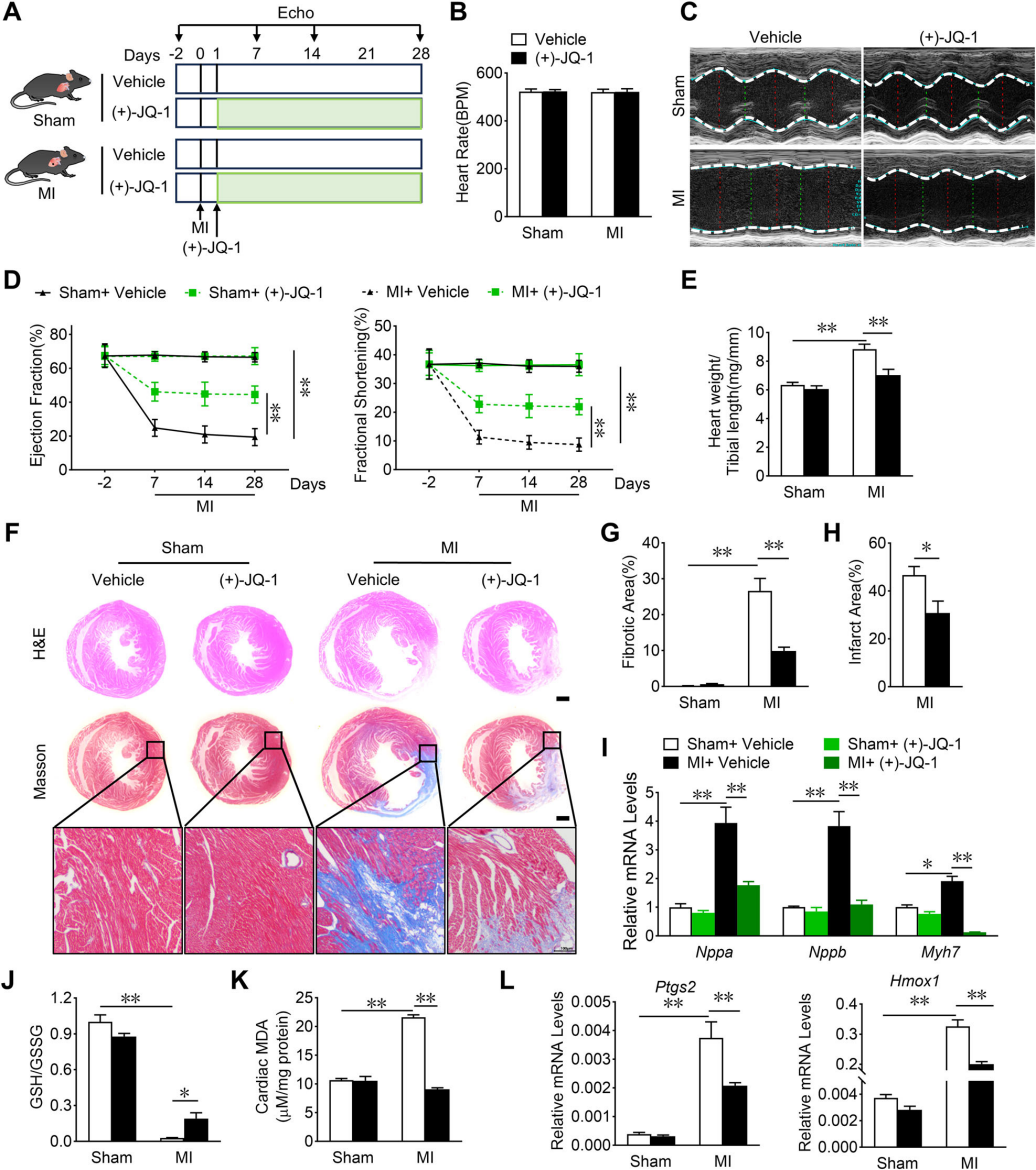
Therapeutic JQ-1 administration improves cardiac remodeling in MI mice. **A** Schematic representation of the protocol for the treatment of MI mice with JQ-1. JQ-1 (50 mg/kg) or equivalent vehicle was injected intraperitoneally 1 day after MI. **B** Heart rate of JQ-1-treated mice after 28 days of MI (n = 6). **C** Representative M-mode echocardiography images in mice 28 days post MI treated with JQ-1. **D** Effect of JQ-1 treatment on LVEF and LVFS at indicated days after MI (n = 6). **E** Heart weight to tibial length ratios in mice 28 days post MI treated with JQ-1 (n = 6). **F** Representative H&E and Masson’s trichrome stained of heart sections from mice 28 days post MI treated with JQ-1. Scale bars, 1 mm. Quantification of the fibrosis area (**G**) and infarct area (**H**) of the myocardium from mice 28 days post MI treated with JQ-1 (n = 6). **I** Effect of JQ-1 treatment on *Nppa*, *Nppb*, and *Myh7* mRNA levels in the cardiac tissue from mice 3 days post-MI (n = 6). The cardiac GSH/GSSG levels (**J**) and MDA levels (**K**) from mice 3 days post-MI (n = 6). **L** Analysis of *Ptgs2* and *Hmox1* mRNA levels of the myocardium in mice 3 day after MI (n = 6). The data are presented as mean ± SEM. The statistical significance of the data was evaluated using a Student’s two-tailed t test (**H**), and two-way ANOVA followed by Tukey’s test for multiple comparisons (**B**, **D**, **E**, **G** and **I**–**L**). *P < 0.05; **P < 0.01.

To further explore whether JQ-1 administration after MI has therapeutic value, mice received intraperitoneal injections of JQ-1 1day after MI (Fig. 4A). Therapeutic JQ-1 administration did not affect the heart rates of the sham and MI mice (Fig. 4B) and had beneficial effects in attenuating cardiac injury and dysfunction in MI mice, as demonstrated by higher LVEF and LVFS values, lower heart weight to tibia length ratios, reduced infarct areas, less interstitial fibrosis, and decreased mRNA levels of cardiac stress genes including *Nppa*, *Nppb*, and *Myh7* (Fig. 4C–I). In addition, therapeutic JQ-1 administration improved the ferroptosis-related indicators in MI, e.g., by increasing the GSH/GSSG ratio and decreasing the MDA levels and the expression of pro-ferroptosis genes in infarcted hearts (Fig. 4J–L). Taken together, these data demonstrate that therapeutic JQ-1 administration 1 day after MI had protective effects on cardiac remodeling after MI in mice.

### JQ-1 inhibits cardiomyocyte ferroptosis through the NAMPT/SIRT1 pathway

**Fig. 5:**
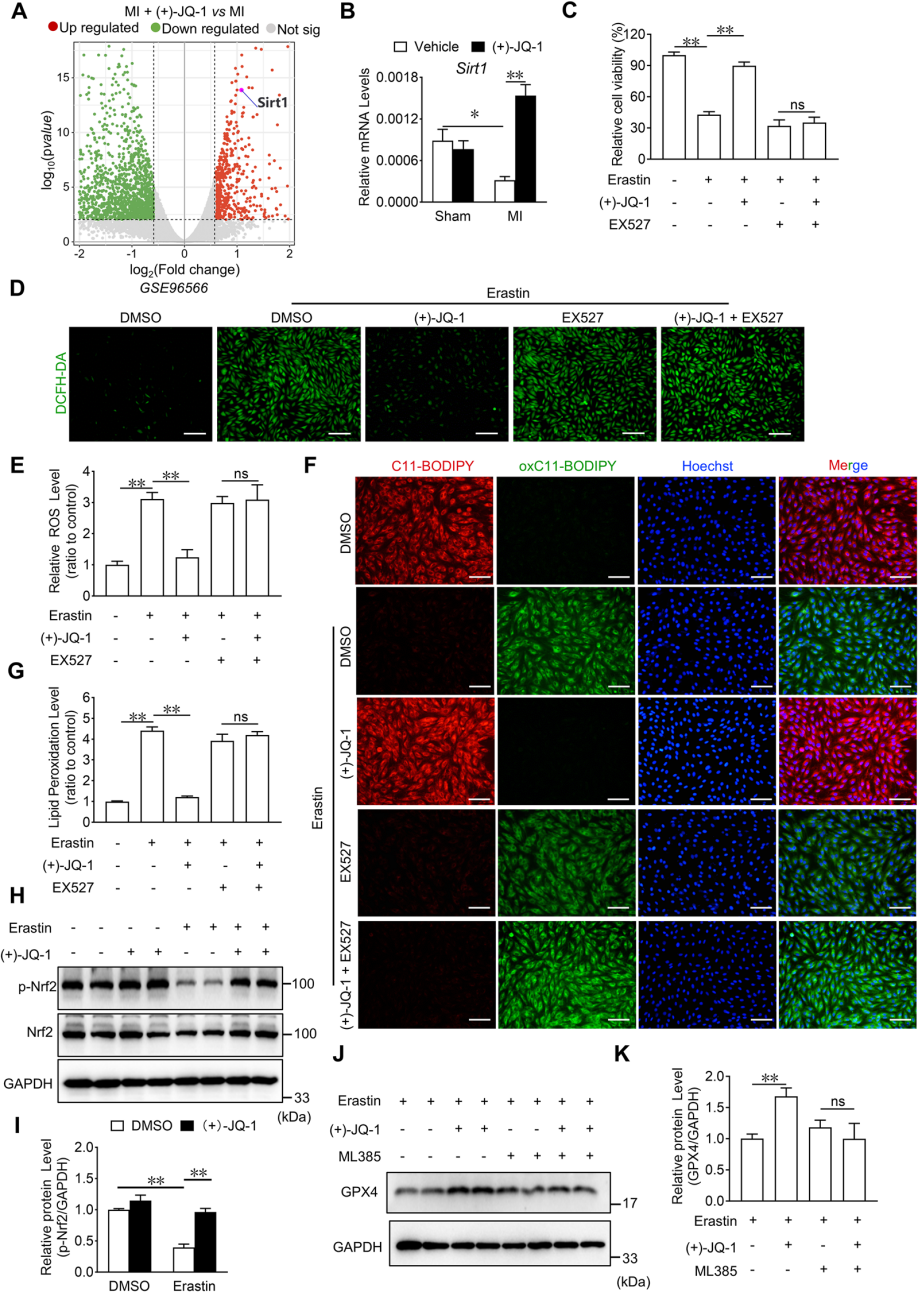
JQ-1 protects cardiomyocytes from ferroptosis by promoting SIRT1 expression. **A** Volcano map of differentially expressed genes in the cardiac tissue from MI with JQ-1 administration and MI in *GSE96566*. Green dots [Log_2_(Fold change) <–0.5 and Log_10_(pvalue) >2] indicate downregulated genes, red dots [Log_2_(Fold change) >0.5 and Log_10_(pvalue) > 2] indicate upregulated genes, gray dots indicate no significant change genes. **B** Effect of JQ-1 treatment on *Sirt1* mRNA levels in the cardiac tissue from mice 1 day after MI (n = 6). **C** Effect of EX527 on the cell viability in H9C2 cardiomyocytes with JQ-1 treatment under the erastin challenge (n = 6). **D** Representative fluorescence images of DCFH-DA staining in H9C2 cardiomyocytes treated with JQ-1 combined with EX527 under the erastin challenge. Scale bars, 100 µm. **E** Quantitative analysis of ROS levels in (**D**) (n = 3). **F** Representative lipid peroxidation images in H9C2 cardiomyocytes treated with JQ-1 combined with EX527 under the erastin challenge. Red: unoxidized C11-BODIPY; Green: oxidized C11-BODIPY; Blue: Hoechst-stained nucleus. Scale bars, 50 μm. **G** Quantitative analysis of lipid peroxidation levels in (**F**) (n = 6). Western blot detection (**H**) and quantification (**I**) of p-Nrf2 and Nrf2 levels in JQ-1-treated H9C2 cardiomyocytes under the erastin challenge (n = 4). Western blot detection (**J**) and quantification (**K**) of GPX4 levels in JQ-1 and/or ML385 treated H9C2 cardiomyocytes under the erastin challenge (n = 4). The data are presented as mean ± SEM. The statistical significance of the data was evaluated using one-way ANOVA with Tukey’s post-hoc test (**C**, **E**, and **G**), and two-way ANOVA followed by Tukey’s test for multiple comparisons (**B**, **I**, and **K**). *P < 0.05; **P < 0.01.

To gain a deeper understanding of the molecular mechanism of by which JQ-1 inhibits ferroptosis in cardiomyocytes, we analyzed RNA-seq data (*GSE96566*) of left ventricular samples showing MI with JQ-1 administration and MI [18]. Among the differentially expressed genes, we identified *Sirt1*, which encodes SIRT1, a NAD^+^-dependent histone deacetylase that plays a pivotal role in numerous pathophysiological processes, including cardiomyocyte ferroptosis (Fig. 5A and Supplementary Fig. 4A, B). We then examined the mRNA expression changes in *Sirt1* in infarcted heart tissues. As expected, the mRNA level of *Sirt1* decreased after MI, whereas the administration of JQ-1 resulted in an increase in *Sirt1* mRNA level (Fig. 5B). EX527, a specific inhibitor of SIRT1, abolished the protective effect conferred by JQ-1 against erastin-induced ferroptosis in H9C2 cells, as demonstrated by increased ROS production and accumulation of lipid peroxidation products in erastin-treated H9C2 cardiomyocytes (Fig. 5C–G). SIRT1 protects cardiomyocytes from ferroptosis via nuclear factor erythroid 2-related factor 2 (Nrf2), which needs to be phosphorylated into the nucleus to exert antioxidant effects [33, 34]. We found that erastin inhibited phosphorylation of Nrf2 (p-Nrf2) and JQ-1 rescued the reduction of p-Nrf2 (Fig. 5H, I). ML385, an inhibitor of Nrf2, abolished the elevation of GPX4 levels by JQ-1 in erastin-treated H9C2 cardiomyocytes (Fig. 5J, K).

**Fig. 6:**
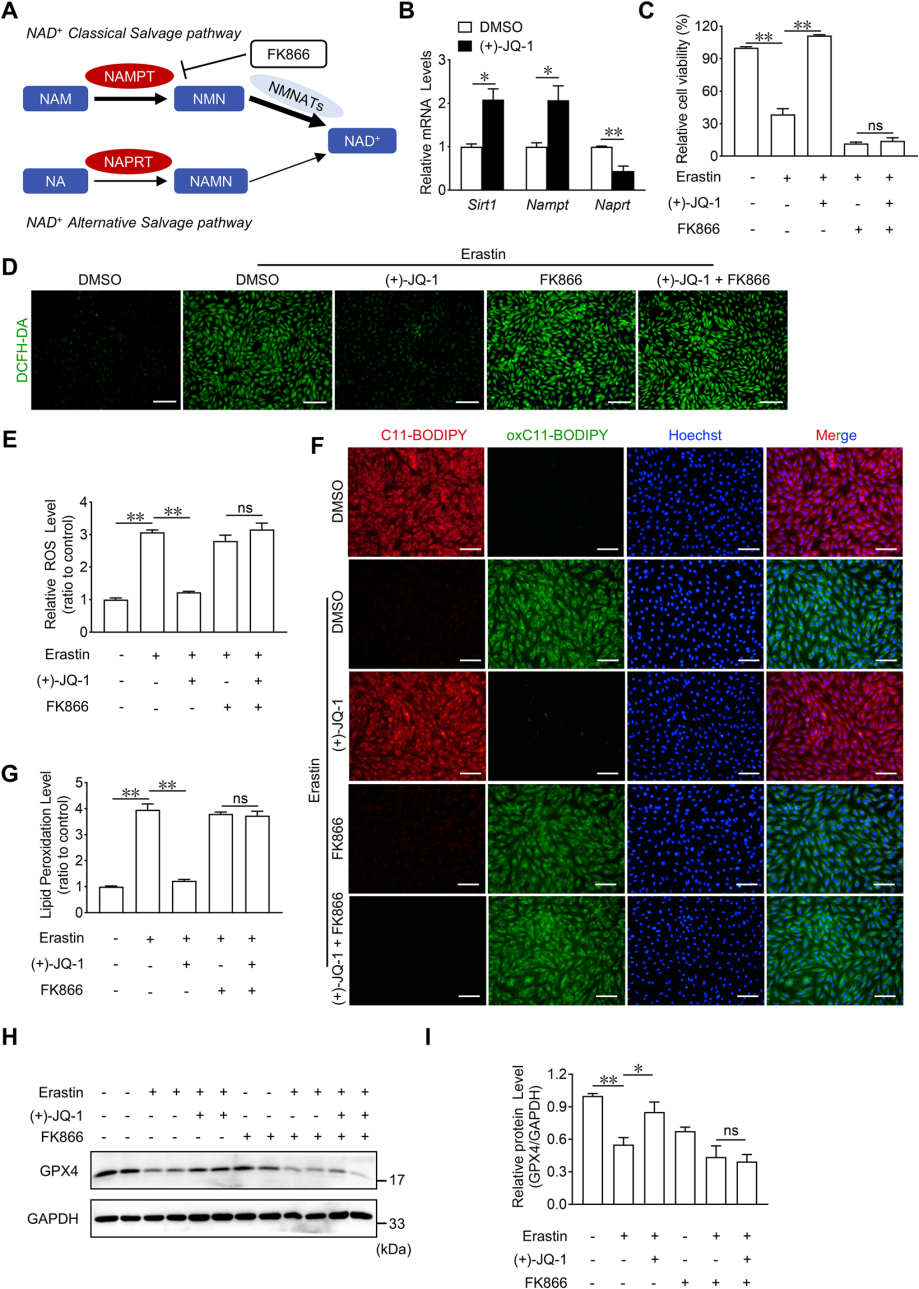
JQ-1 inhibits erastin-induced ferroptosis by activating the NAMPT-SIRT1 signaling. **A** Schematic illustration of NAD^+^ synthesis pathway in cardiomyocytes, FK866 is the inhibitor of NAMPT. **B** Relative mRNA levels of *Sirt1*, *Nampt*, and *Naprt* in H9C2 cardiomyocytes treated with JQ-1 for 24 h (n = 3). **C** Effect of FK866 on the cell viability in H9C2 cardiomyocytes with JQ-1 treatment under the erastin challenge (n = 6). **D** Representative fluorescence images of DCFH-DA staining in H9C2 cardiomyocytes treated with JQ-1 combined with FK866 under the erastin challenge. Scale bars, 100 µm. **E** Quantitative analysis of ROS levels in (**D**) (n = 3). **F** Representative lipid peroxidation images in H9C2 cardiomyocytes treated with JQ-1 combined with FK866 under the erastin challenge. Red: unoxidized C11-BODIPY; Green: oxidized C11-BODIPY; Blue: Hoechst-stained nucleus. Scale bars, 50 μm. **G** Quantitative analysis of lipid peroxidation levels presented in (**F**) (n = 6). **H** Western blot analysis of the effect of FK866 on the protein levels of GPX4 in H9C2 cardiomyocytes with JQ-1 treatment under the erastin challenge. **I** Quantitative analysis of the relative GPX4 protein levels of in (**H**) (n = 6). The data are presented as mean ± SEM. The statistical significance of the data was evaluated using one-way ANOVA with Tukey’s post-hoc test (**C**, **E**, **G**, and **I**). *P < 0.05; **P < 0.01.

As a cofactor for SIRT1, intracellular NAD^+^ biosynthesis is mainly performed through NAMPT and NAPRT-mediated pathways [35] (Fig. 6A). Inhibition of BRD4 has been reported to suppress the expression of NAPRT and promote the expression of NAMPT [36]. Interestingly, JQ-1 treatment resulted in an increase in mRNA levels of *Nampt* and *Sirt1*, accompanied by a decrease in the mRNA level of *Naprt* in H9C2 cardiomyocytes, regardless of whether erastin challenge or not (Fig. 6B and Supplementary Fig. 5A). In fact, RNA-seq data (*GSE96566*) and our RT-PCR analysis also showed that JQ-1 administration decreased the mRNA levels of *Naprt* and increased the mRNA levels of *Nampt* and *sirt1* in the infarcted heart (Supplementary Fig. 5B, C and Fig. 5B). ChIP assays demonstrated that BRD4, a direct target of inhibited by JQ-1, binds to the promoter region of the *Naprt* gene (Supplementary Fig. 5D). In addition, JQ-1 reversed the erastin-induced reduction of NAD^⁺^ levels (Supplementary Fig. 5E). Moreover, FK866, a potent and specific inhibitor of NAMPT, abolished the protective effect of JQ-1 against erastin-induced ferroptosis by increasing the production of ROS and accumulation of lipid peroxidation products in H9C2 cardiomyocytes (Fig. 6C-G). Additionally, FK866 abolished the JQ-1-induced increase in p-Nrf2 and GPX4 protein levels in erastin-treated H9C2 cardiomyocytes (Supplementary Fig. 5F, G, and Fig. 6H, I). Together, these findings suggest that JQ-1 administration activates the NAMPT/SIRT1 signaling cascade, thereby attenuating erastin-induced ferroptosis in cardiomyocytes.

### JQ-1-PROTAC inhibits erastin-induced ferroptosis in H9C2 cardiomyocytes

**Fig. 7:**
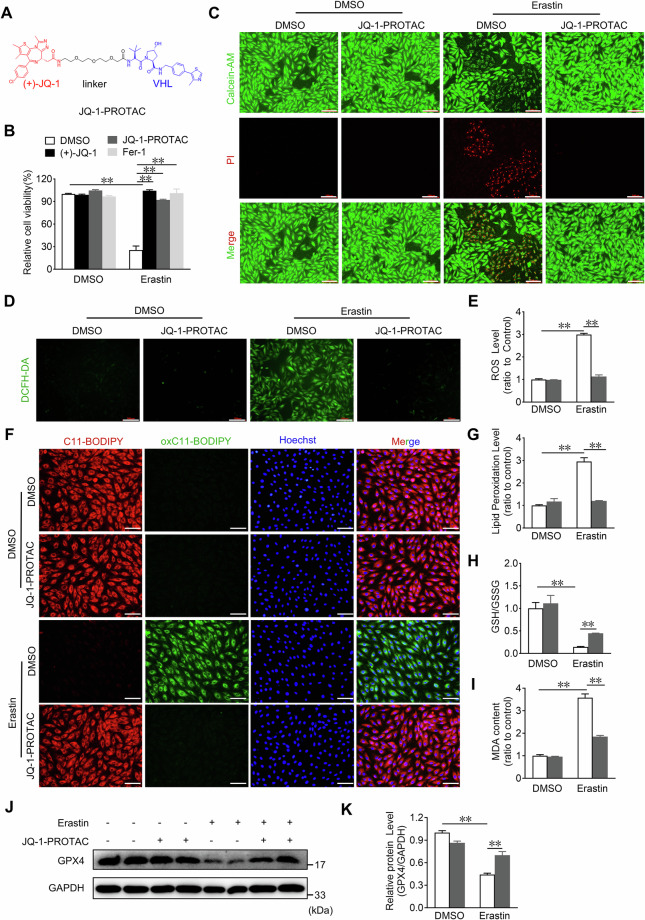
JQ-1-PROTAC reduces erastin-induced death in H9C2 cardiomyocytes. **A** JQ-1-PROTAC Chemical formula. The Figure was created with ChemDraw software (https://www.chemdraw.com.cn/). **B** Effect of JQ-1-PROTAC (0.5 μM) on the cell viability in H9C2 cardiomyocytes under the erastin challenge. JQ-1 and Fer-1 were used as positive controls (n = 6). **C** Representative images of Calcein-AM and PI staining in H9C2 cardiomyocytes treated with JQ-1-PROTAC under the erastin challenge. Green indicates the fluorescence of Calcein-AM, red indicates the fluorescence of PI. Scale bars, 100 µm, (n = 6). **D** Representative DCFH-DA fluorescence (ROS probe) images in H9C2 cardiomyocytes treated with JQ-1-PROTAC under the erastin challenge. Scale bars, 100 µm. **E** Quantitative analysis of ROS levels in (**D**) (n = 4). **F** Representative lipid peroxidation staining images in H9C2 cardiomyocytes treated with JQ-1-PROTAC under the erastin challenge. Red: unoxidized C11-BODIPY; Green: oxidized C11-BODIPY; Blue: Hoechst-stained nucleus. Scale bars, 50 μm. **G** Quantitative analysis of lipid peroxidation levels in (F) (n = 3). Quantitative analysis of GSH/GSSG levels (**H**) and MDA levels (**I**) in H9C2 cardiomyocytes with JQ-1-PROTAC treatment under the erastin challenge (n = 3). **J** Western blot analysis of the GPX4 protein levels in H9C2 cardiomyocytes with JQ-1-PROTAC treatment under the erastin challenge. **K** Quantitative analysis of relative GPX4 protein levels in (**J**) (n = 4). The data are presented as mean ± SEM. The statistical significance of the data was evaluated using a two-way ANOVA followed by Tukey’s test for multiple comparisons (**B**, **E**, **G**–**I**, and **K**). **P < 0.01.

PROTACs, an emerging technology that catalyzes the degradation of target proteins by hijacking the endogenous ubiquitination system, have shown great potential as a new therapeutic approach for various diseases [37]. To provide an alternative application of JQ-1 in the inhibition of cardiomyocyte ferroptosis, we treated cardiomyocytes with our previously developed JQ-1-PROTAC [38] (Fig. 7A). Similar to the findings of previous reports, we observed that JQ-1-PROTAC promoted BRD4 degradation in H9C2 cardiomyocytes (Supplementary Fig. 6). JQ-1-PROTAC inhibited erastin-induced cell death in H9C2 cardiomyocytes (Fig. 7B) and significantly restored the reduced fluorescence intensity of Calcein AM in the erastin-challenged H9C2 cells (Fig. 7C). Treatment with JQ-1-PROTAC also attenuated erastin-induced cell ferroptosis (Fig. 7C) by reducing the production of ROS (Fig. 7D, E) and accumulation of lipid peroxidation products (Fig. 7F, G) in erastin-exposed H9C2 cardiomyocytes. Consistent with these findings, JQ-1-PROTAC treatment increased the GSH/GSSG ratio, decreased the MDA levels, and increased the protein levels of GPX4 (Fig. 7H–K). Taken together, these results indicate that JQ-1-PROTAC exhibited similar protection against erastin-induced cardiomyocyte ferroptosis as JQ-1.

### JQ-1-PROTAC impedes erastin-induced ferroptosis in human cardiomyocytes

**Fig. 8:**
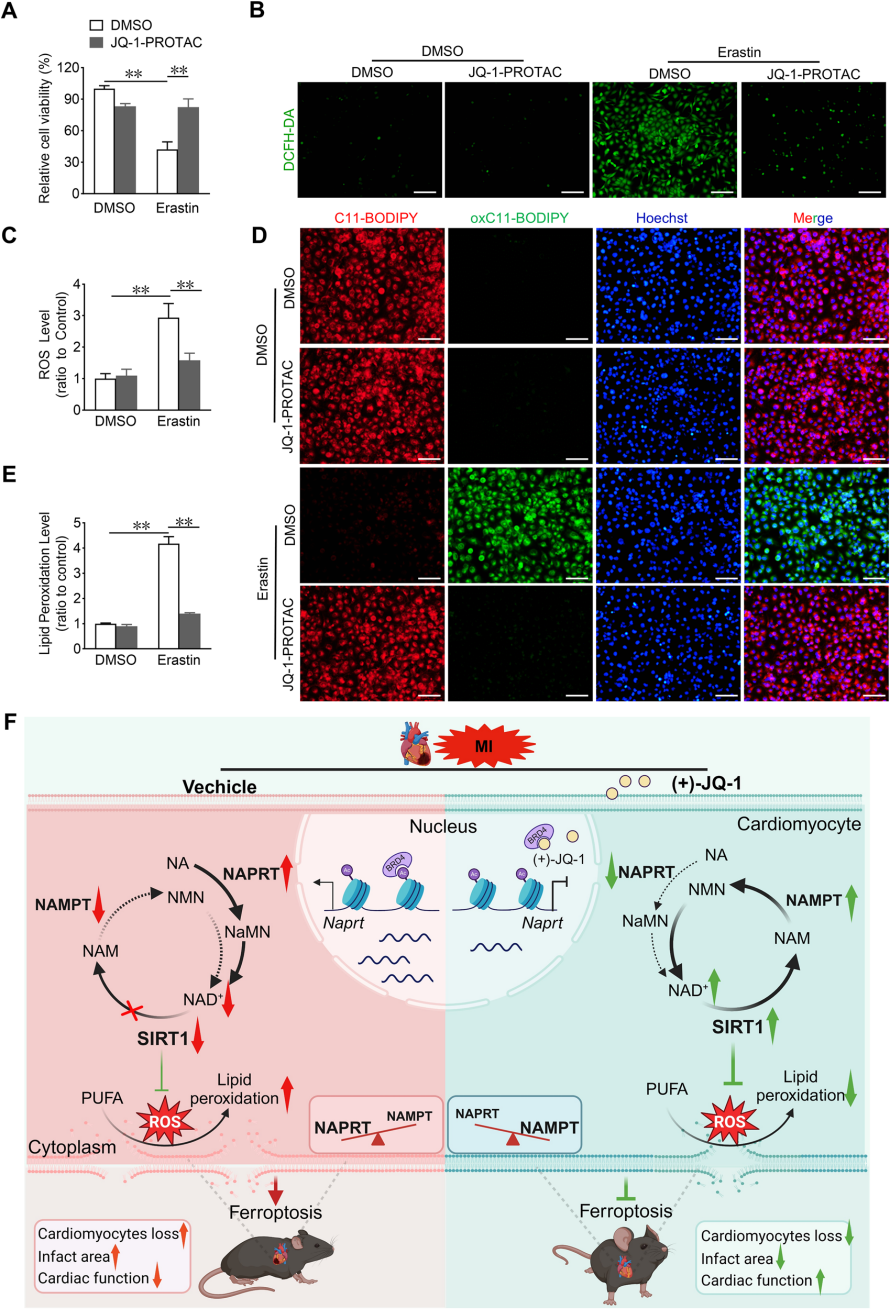
JQ-1 reduces erastin-induced death in human cardiomyocytes. **A** Effect of JQ-1-PROTAC on the cell viability in AC16 cardiomyocytes under the erastin challenge (n = 6). **B** Representative fluorescence images of DCFH-DA staining in AC16 cardiomyocytes treated with JQ-1-PROTAC under the erastin challenge. Scale bars, 100 µm. **C** Quantitative analysis of ROS levels in (**B**) (n = 3). **D** Representative lipid peroxidation images in AC16 cardiomyocytes treated with JQ-1-PROTAC under the erastin challenge. Red: unoxidized C11-BODIPY; Green: oxidized C11-BODIPY; Blue: Hoechst-stained nucleus. Scale bars, 50 μm. **E** Quantitative analysis of lipid peroxidation levels in (**D**) (n = 3). **F** Schematic diagram of JQ-1 protects against MI-induced cardiomyocytes ferroptosis through NAMPT-SIRT1 signaling. NA nicotinic acid, NAPRT NA phosphoribosyltransferase, NaMN nicotinic acid mononucleotide, NAD^+^ nicotinamide adenine dinucleotide, NAM nicotinamide, NMN nicotinamide mononucleotide, NAMPT nicotinamide phosphoribosyl-transferase, PUFA polyunsaturated fatty acid. Created with BioRender.com. The data are presented as mean ± SEM. The statistical significance of the data was evaluated using a two-way ANOVA followed by Tukey’s test for multiple comparisons (**A**, **C**, and **E**). **P < 0.01.

Finally, to ascertain the potential clinical applications of JQ-1-PROTAC, we investigated the impact of JQ-1-PROTAC treatment on erastin-induced ferroptosis in AC16 human cardiomyocytes. Similar to the findings obtained in H9C2 cells, treatment with JQ-1-PROTAC markedly increased in the reduced cell viability of erastin-treated AC16 cardiomyocytes (Fig. 8A), and markedly reduced erastin-induced ROS production and accumulation of lipid peroxidation products in AC16 cardiomyocytes (Fig. 8B–E). These findings indicate that JQ-1-PROTAC has the potential to be an efficacious agent for treating ferroptosis-related heart disease.

## Discussion

Cardiomyocyte death is a crucial factor in cardiac remodeling after MI, and effective reduction of cardiomyocyte death remains a research hotspot for improving the prognosis of MI [8]. In this study, we identified JQ-1 as a promising small-molecule compound that inhibited erastin-induced ferroptosis in cardiomyocytes by screening a library of epigenetic compounds and conducting biochemical, molecular, and cellular experiments. Both prophylactic and therapeutic JQ-1 administration effectively attenuated cardiac injury and dysfunction by inhibiting ferroptosis via the NAMPT/SIRT1 pathway. Importantly, JQ-1-PROTAC potently inhibited ferroptosis in H9C2 and AC16 cardiomyocytes, which providing a novel alternative approach for the potential application of JQ-1. Thus, JQ-1 is a promising small-molecule compound for the prevention and treatment of ferroptosis in cardiomyocytes after MI (Fig. 8F).

Among the 773 epigenetic target compounds, we identified the target molecule JQ-1 using an erastin-induced ferroptosis model, CCK-8 assay, and a literature review. JQ-1 was the first and most well-characterized BRD4 inhibitor reported in 2010, and it was shown to replace the BRD4 protein on chromatin by competitive binding to the acetyl-lysine-binding pocket of BRD4 bromodomains [39]. Because of its good cell membrane penetration and potent inhibitory effects at a low concentration, JQ-1 has been extensively studied for its anti-tumor, anti-organ fibrosis, and anti-inflammatory activities [39–42]. We found that JQ-1 treatment notably suppressed erastin-induced ferroptosis in cardiomyocytes and improved cardiac remodeling after MI in mice. However, in a variety of cancer lines, JQ-1 has been reported to promote erastin-induced ferroptosis by downregulating a number of genes associated with this process, such as *SLC7A11*, *SLC3A2*, and *GPX4* [43, 44]. Consistent with our observations, inhibition of BRD4 by JQ-1 suppressed erastin-induced ferroptosis in CALU1 and HT1080 cells and abrogated the anti-tumor effects conferred by erastin in a lung metastasis model by protecting mitochondrial function and reducing lipid ROS formation [45]. Interestingly, JQ-1 has been shown to induce ferroptosis in senescent human dermal fibroblasts by reducing the expression of ferroptosis resistance genes in senescent cells, but has no effect on non-senescent cells [46]. These seemingly contradictory results suggest that the role of JQ-1 differs across different types of cells. Although much of the existing research indicates that JQ-1 administration is beneficial for a variety of diseases, further studies are required to elucidate the exact role of JQ-1 in different cell types and cells of different ages.

JQ-1 has been reported to be beneficial in many cardiovascular diseases by reducing atherosclerosis, inhibiting intimal neogenesis, and alleviating cardiac hypertrophy [17, 18, 47–49]. JQ-1 administration suppresses phenylephrine-induced cellular hypertrophy in NRVMs and inhibits transverse aortic constriction-induced pathological cardiac hypertrophy and heart failure in mice [17]. Daily injection of JQ-1 has been shown to improve cardiac dysfunction caused by the deletion of Lmna in the cardiomyocytes of mice, abrogate cardiac arrhythmia, fibrosis, and cardiomyocyte apoptosis, and prolong the survival time of mice [50]. In a rat MI model, JQ-1 treatment mitigated MI-induced damage and improved cardiac function injury by suppressing TLR4 signaling activation [21]. Although JQ-1 has been previously reported to alleviate cardiac remodeling after MI in mice, the JQ-1 intervention in that study was performed six days after MI, and a large MI model was used [18]. In contrast, we found that treatment with JQ-1 before MI or 1 day after MI significantly improved cardiac remodeling. The collective findings indicate that JQ-1 is a promising small-molecule compound for the improving cardiac function after MI.

NAD^+^ and its metabolites play important regulatory roles in basal and stress processes, and are involved in a multitude of cellular events, including redox reactions, energy metabolism, senescence, and inflammation [35]. Both in human failing hearts and in pathologically hypertrophied mouse hearts, an increased NADH/NAD^+^ ratio promotes protein hyperacetylation and mitochondrial dysfunction, and normalization of NADH/NAD^+^ imbalances inhibits protein hyperacetylation and improves cardiac function under stress [51]. In addition to being a coenzyme for redox reactions, NAD^+^ is an important cofactor for non-redox NAD^+^-dependent enzymes such as sirtuins [35]. NAD^+^-dependent SIRT1 is essential for the maintenance of normal heart function and a moderate increase in SIRT1 levels has been shown to retard cardiac aging by attenuating cardiac hypertrophy, apoptosis/fibrosis, and cardiac dysfunction [52]. Owing to the lack of the enzyme required for de novo synthesis of NAD^+^ from tryptophan in the heart, NAD^+^ in cardiomyocytes is synthesized through the salvage biosynthesis pathway of two key enzymes, namely, NAMPT and NAPRT, with NAMPT-mediated synthesis accounting for the majority [53]. In the present study, we observed that JQ-1 administration enhanced the decline in NAMPT expression in infarcted hearts and reduced ferroptosis in cardiomyocytes. In fact, increasing levels of NAD^+^ have been shown to improve many forms of heart failure [53], and cardiac-specific overexpression of NAMPT in transgenic mice has been shown to reduce infarct size and promote cardiomyocyte survival by preventing the decrease in NAMPT and increasing NAD^+^ content post-MI [54].

In summary, we found that administration of the small-molecule compound JQ-1 improved cardiac remodeling after MI by inhibiting ferroptosis through the NAMPT/SIRT1 pathway, indicating a novel potential strategy for the intervention of ferroptosis-related cardiomyopathy.

## Supplementary information

Supplementary materials

uncropped original western blots

## Data Availability

All data generated or analyzed during this study are included in this article and its supplementary information files. The data supporting the findings of this study are available from the corresponding author upon reasonable request.
