# Supplementary material for: (+)-JQ-1 alleviates cardiac injury in myocardial infarction by inhibiting ferroptosis through the NAMPT/SIRT1 pathway
Source: Cell Death Dis. 2025 Jul 23;16(1):548. doi: 10.1038/s41419-025-07880-x (PMC12283960; doi:10.1038/s41419-025-07880-x)
Supplement: Supplementary file 1 — Supplementary materials [file 41419_2025_7880_MOESM1_ESM.docx]

**Supplementary materials**

**(+)-JQ-1 alleviates cardiac injury in myocardial infarction by inhibiting ferroptosis through the NAMPT/SIRT1 pathway**

Mengxue Yang^1,5^, Ting Wang^1,5^, Jingrong Shao^1,5^, Xinna Ran^1^, Rui Xiao^1^, Rui Zhao^2^, Chunyan Wu^1^, Ming Ji^3^, Weiping Tian^2^, Huabing Sun^3^, Jiao Liu^4,🖂^, Shengkai Zuo^1,🖂^

^1^Department of Biopharmaceutics, Tianjin Key Laboratory of Technologies Enabling Development of Clinical Therapeutics and Diagnostics, Key Laboratory of Immune Microenvironment and Disease (Ministry of Education), The Province and Ministry Co-sponsored Collaborative Innovation Center for Medical Epigenetics, School of Pharmacy, Tianjin Medical University, Tianjin, China.

^2^Research Center of Basic Medical Science, Tianjin Medical University, Tianjin, China.

^3^Department of Chemical Biology, School of Pharmacy, Tianjin Medical University, Tianjin, China.

^4^Department of Pharmacology, Tianjin Key Laboratory of Inflammatory Biology, School of Basic Medical Sciences, Tianjin Medical University, Tianjin, China.

^5^These authors contributed equally: Mengxue Yang, Ting Wang, and Jingrong Shao.

^🖂^E-mail：[drliujiao@tmu.edu.cn](mailto:drliujiao@tmu.edu.cn); [zuoshengkai@tmu.edu.cn](mailto:zuoshengkai@tmu.edu.cn)

**Supplementary Figure 1-6**

**Supplementary Table 1-3**

**
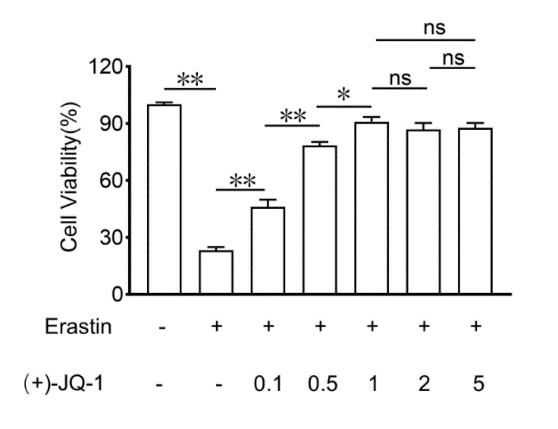
**

**Supplementary Fig. 1 Effect of JQ-1 on erastin-induced ferroptosis in H9C2 cardiomyocytes.**

Effect of different concentrations of JQ-1 on the cell viability of H9C2 cardiomyocytes (n = 6). Data represent mean ± SEM. Statistical significance was evaluated with one-way ANOVA with Tukey’s post-hoc test. **P* < 0.05, ***P* < 0.01.


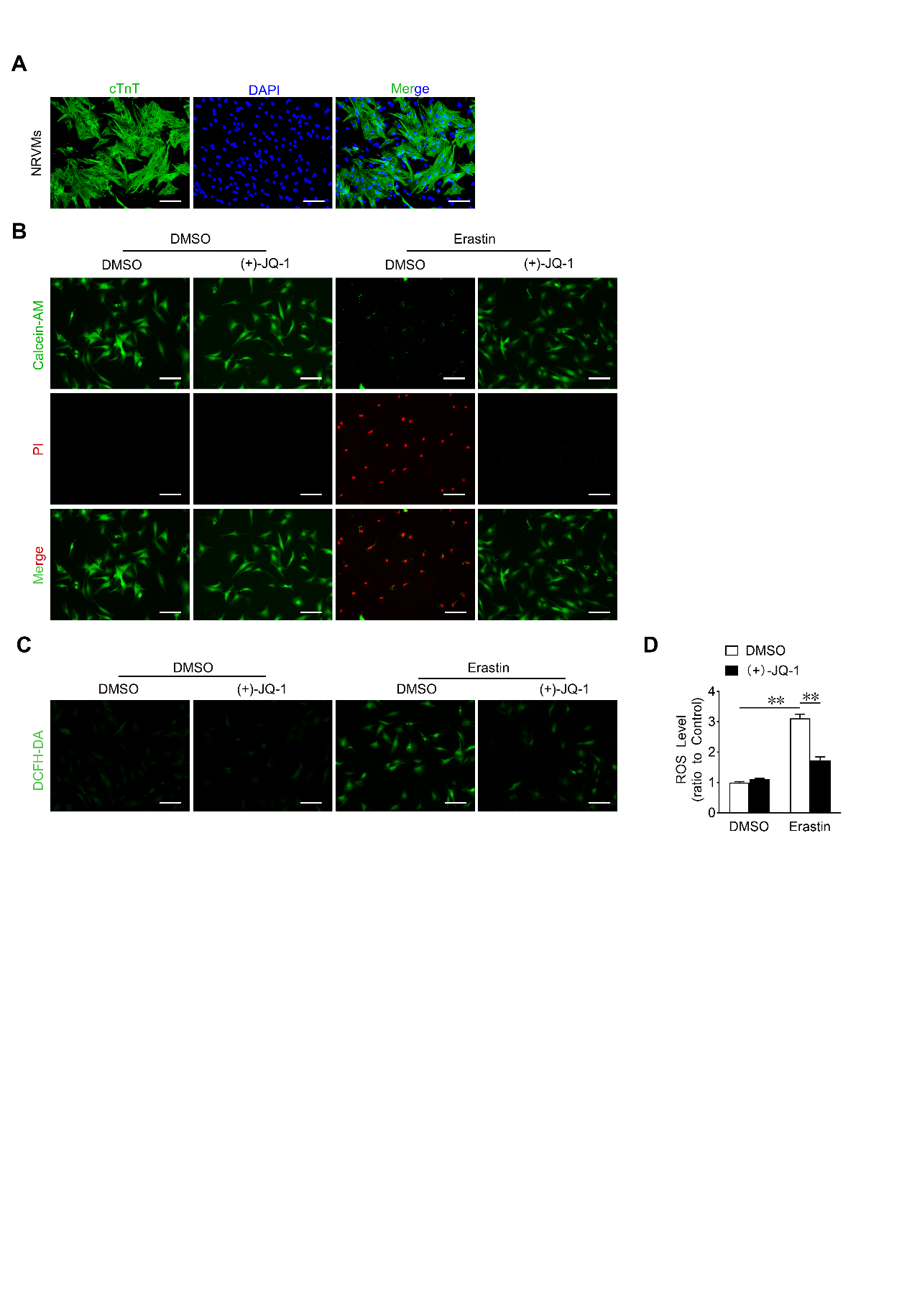


**Supplemental Fig. 2 JQ-1 reduces cell death and ROS levels in NRVMs after erastin treatment.** **A** Immunofluorescence staining of cardiac troponin T (cTnT) in NRVMs. Green: cTnT, blue: DAPI. Scale bars, 50 µm. **B** The representative images of Calcein-AM and propidium iodide (PI) staining in NRVMs treated with JQ-1 under the erastin challenge. Green: Calcein-AM, red: PI. Scale bars, 100 µm (n = 6). **C** Representative DCFH-DA fluorescence images in NRVMs treated with JQ-1 under the erastin challenge. Scale bars, 100 µm. **D** Quantitative analysis of ROS levels in NRVMs (n = 6). Data represent mean ± SEM. Statistical significance was evaluated with two-way ANOVA followed by Tukey’s test. ***P* < 0.01.

**
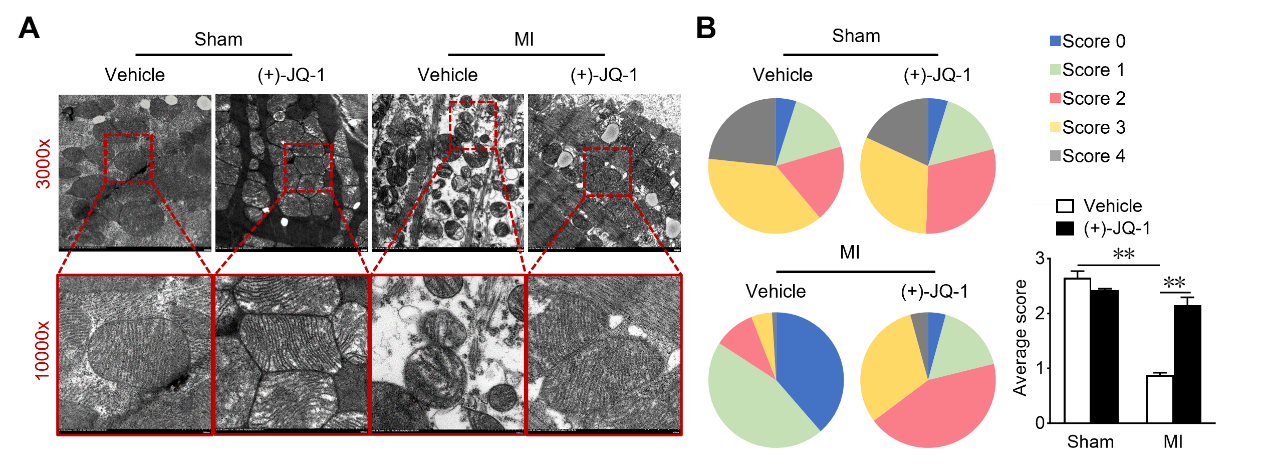
**

**Supplementary Fig. 3**  **JQ-1 treatment improves mitochondrial morphological abnormalities caused by MI. A** Representative transmission electron microscopy images of heart section from mice 1 day after MI. **B** A 5-grade scoring system was used to evaluate the score distribution and average score of cardiomyocyte mitochondria. Score 4, the mitochondrial cristae content >80%, the cristae are well-defined and clear; score 3, the mitochondrial cristae content 60%-80%, the cristae are slightly irregular; score 2, the mitochondrial cristae content 30%-60%; score 1, the mitochondrial cristae content 10%-30%, cristae are severely fragmented or swollen and distorted membranes; score 0, the mitochondrial cristae content <10% and severely disrupted membranes. Per heart, 20 to 40 mitochondria (10000×) were analyzed (n = 3). Data represent mean ± SEM. Statistical significance was evaluated using a two-way ANOVA followed by Tukey’s test for multiple comparisons. ***P* < 0.01.

**
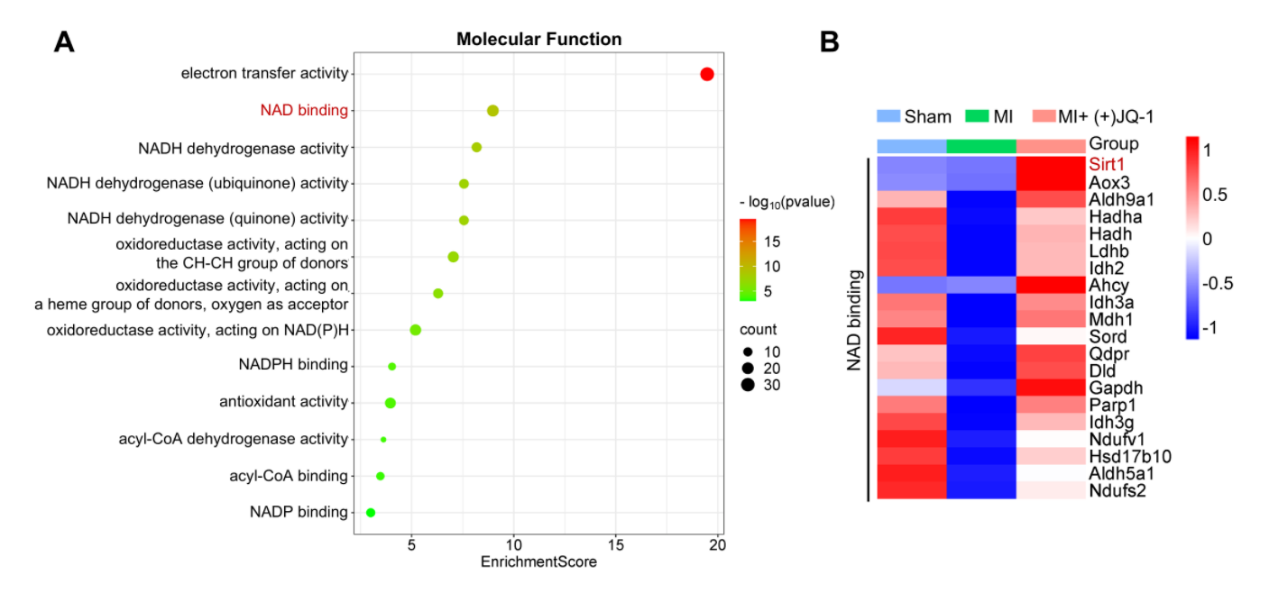
**

**Supplementary Fig. 4**  **Effect of JQ-1 on the gene transcriptome after MI. A** Gene Ontology (GO) enrichment analyses of signaling pathways associated with differentially expressed genes between MI with JQ-1 administration and MI. **B** The heatmap of the NAD^+^ binding related genes in RNA-seq between sham, MI and MI with JQ-1 administration. Gene expressions were normalized with row Z-score.

**
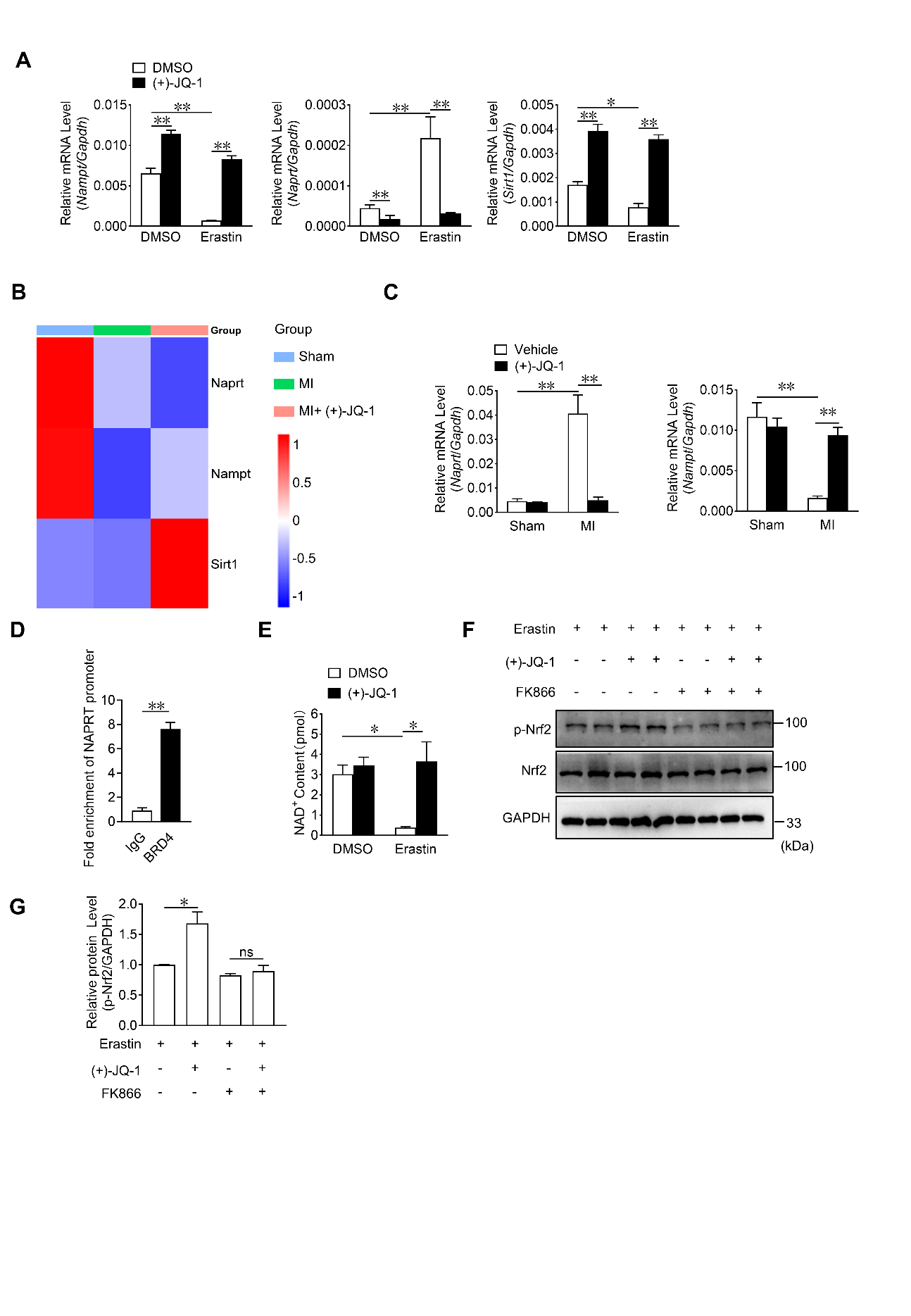
**

**Supplementary Fig. 5 Effect of JQ-1 on mRNA levels of erastin-induced NAD^+^-related genes. A** Relative mRNA levels of *Nampt*, *Naprt*, and *Sirt1* in H9C2 cardiomyocytes treated with JQ-1 in the presence of erastin (n = 3). **B** Representative heatmap of the synthesis and utilization of NAD^+^-related genes between sham, MI and MI with JQ-1 administration. Genes expression were normalized with row Z-score. **C** Effect of JQ-1 treatment on *Naprt* and *Nampt* mRNA levels in the cardiac tissue from mice 1 day after MI (n = 6). **D** ChIP-PCR was utilized to detect the binding of BRD4 to the promoter of NAPRT in H9C2 cardiomyocytes (n = 3). **E** Effect of JQ-1 treatment on NAD^+^ levels in H9C2 cardiomyocytes treated with JQ-1 under the erastin challenge (n = 3). **F** Western blot analysis of the effect of FK866 on the protein levels of p-Nrf2 and Nrf2 in H9C2 cardiomyocytes with JQ-1 treatment under the erastin challenge. **G** Quantitative analysis of the relative p-Nrf2 protein levels of in (F) (n = 4). Data represent mean ± SEM. Statistical significance was evaluated with two-way ANOVA followed by Tukey’s test for multiple comparisons (A, C, E and G) and Student's t test (D). **P* < 0.05, ***P* < 0.01.

**
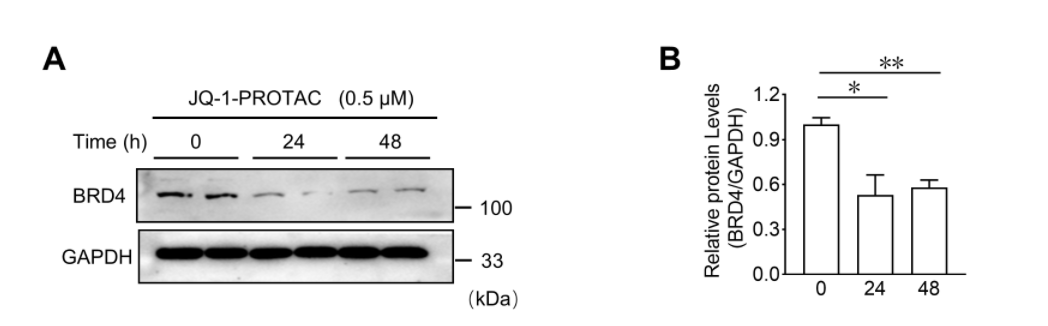
**

**Supplementary Fig. 6**  **Effect of JQ-1-PROTAC on BRD4 expression in H9C2 cells.** **A** Western blot analysis of BRD4 protein levels in H9C2 cardiomyocytes after 24 and 48 h of JQ-1-PROTAC treatment. **B** Quantification of relative the protein levels of BRD4 in (A) (n = 4). Data represent mean ± SEM. Statistical significance was evaluated with one-way ANOVA with Tukey’s post-hoc test. **P* < 0.05; ***P* < 0.01.

**Supplementary Table 1. 115 candidate compounds for inhibiting erastin-induced ferroptosis in cardiomyocytes**

| \| MOLENAME \| Log_2_(fold change) \| Receptor \| \| --- \| --- \| --- \| |  |  |
| --- | --- | --- | --- | --- | --- |
| \| Remodelin hydrobromide \| 6.31 \| acetyl-transferase NAT10 \| \| --- \| --- \| --- \| \| β-thujaplicin \| 6.28 \| ERα \| \| Icaritin \| 6.22 \| JAK2; STAT3 \| \| Remodelin \| 6.14 \| NAT10 \| \| Mollugin \| 5.99 \| HER2; JAK2 \| \| Dorsomorphin dihydrochloride \| 5.93 \| AMPK \| \| ZL0420 \| 5.83 \| BRD4 \| \| ACY738 \| 5.79 \| HDAC6 \| \| Birabresib \| 5.77 \| BRDs \| \| C273-0107 \| 5.74 \| Ataxin-2 \| \| J147 \| 5.73 \| BDNF \| \| Hydroxy Camptothecine \| 5.72 \| Topo I \| \| 6-BIO \| 5.67 \| CDK1/CyclinB; CDK2/CyclinA; CDK5/p35; GSK-3; Tyk2 \| \| Mivebresib \| 5.67 \| BET \| \| Pim1/AKK1-IN-1 \| 5.58 \| Pim1; MST2 \| \| Irinotecan \| 5.57 \| Topo I \| \| C794-1675 \| 5.57 \| Lysine-specific demethylase 4A \| \| FL411 \| 5.56 \| BRD4 \| \| SIS17 \| 5.48 \| HDAC11 \| \| BRD4770 \| 5.46 \| G9a \| \| Pyrazolanthrone \| 5.46 \| Aurora A; JNK1; JNK2; JNK3; TrkA \| \| A366 \| 5.46 \| G9a/GLP \| \| KG-501 \| 5.45 \| *CREB* \| \| XL019 \| 5.44 \| FLT3; JAK1; JAK2; JAK3; PDGFRβ \| \| WHI-P131 \| 5.42 \| JAK3; JAK3 \| \| AG490 \| 5.39 \| EGFR; HER2/ErbB2; JAK2 \| \| Tenovin3 \| 5.39 \| SIRT2 \| \| PTACH \| 5.38 \| HDAC \| \| CCT241736 \| 5.36 \| FLT3; Aurora Kinase \| \| MC1568 \| 5.36 \| HD1-A (Maize); HD1-B (Maize) \| \| Rucaparib \| 5.35 \| PARP1 \| \| nAS-E \| 5.34 \| *CREB* \| \| AMI1 \| 5.32 \| PRMT1; yeast Hmt1p \| \| AZD5153 6-Hydroxy-2-naphthoic acid \| 5.31 \| BD1-BRD4; FL-BRD4 \| \| Reversine \| 5.29 \| A3; Aurora A; Aurora B; Aurora C \| \| JNJ7706621 \| 5.27 \| CDK2/CyclinE, CDK2/CyclinA , CDK1/CyclinB , Aurora A , Aurora B \| \| Inauhzin \| 5.25 \| SIRT1 \| \| SRT1720 \| 5.22 \| SIRT1 \| \| Trichostatin A \| 5.20 \| HDAC \| \| 2-Methoxyestradiol \| 5.19 \| HIF-1α; HIF-2α; Microtubule depolymerization \| \| CXD-101 \| 5.17 \| HDAC;HDAC1 ;HDAC3 ;HDAC2 \| \| SF2523 \| 5.17 \| DNA-PK; BRD4; mTOR; PI3Kα; PI3Kγ \| \| Oltipraz \| 5.17 \| Reverse Transcriptase;HIF-1α \| \| BMH21 \| 5.16 \| RNA polymerase I \| \| GSK J4 \| 5.15 \| JMJD3 \| \| Amodiaquine hydrochloride \| 5.15 \| histamine N-methyl transferase \| \| Carvedilol \| 5.15 \| Adrenergic Receptor; HIF; E-selectin; NADPH; Vcam; Potassium Channel; VEGFR \| \| CREB inhibitor \| 5.14 \| CREB \| \| Quisinostat \| 5.14 \| HDAC1; HDAC10; HDAC11; HDAC2; HDAC3; HDAC4; HDAC5; HDAC8 \| \| Vorinostat \| 5.13 \| HDAC; HDAC1;HDAC2;HDAC3;HDAC6;HDAC8 \| \| GSK-J4 Hydrochloride \| 5.07 \| JMJD3 \| \| INCB057643 \| 5.03 \| BET \| \| Belinostat \| 5.01 \| HDAC \| \| CCT137690 \| 5.00 \| Aurora A; Aurora B; Aurora C \| \| SN38 \| 5.00 \| Topo I \| \| Antrapurol \| 4.99 \| AMPK \| \| SNS-314 Mesylate \| 4.98 \| Aurora A; Aurora B; Aurora C \| \| Momelotinib \| 4.95 \| JAK1; JAK2; JAK3 \| \| Rocilinostat \| 4.92 \| HDAC1; HDAC2; HDAC3; HDAC6; HDAC8 \| \| Histone Deacetylase Inhibitor III \| 4.91 \| HDAC \| \| SP2509 \| 4.90 \| LSD1 \| \| KC7F2 \| 4.85 \| HIF-1α \| \| PCI24781 \| 4.85 \| HDAC1; HDAC10; HDAC2; HDAC3/SMRT; HDAC6 \| \| NSC 3852 \| 4.83 \| HDAC \| \| WHI-P154 \| 4.80 \| EGFR; JAK3; Src; VEGFR \| \| Mocetinostat \| 4.71 \| HDAC1; HDAC11; HDAC2; HDAC3; HDAC4 \| \| 5-Azacytidine \| 4.70 \| DNA methyltransferase (DNMT) \| \| Sotrastaurin \| 4.66 \| PKCα; PKCβ1; PKCδ; PKCη; PKCθ \| \| 4SC-202 tosylate \| 4.64 \| HDAC1;HDAC2;HDAC3;HDAC5;HDAC9;HDAC10;HDAC11; \| \|  \| 4.64 \| HDAC1; HDAC10; HDAC11; HDAC2; HDAC3; HDAC4; HDAC5; HDAC6; HDAC7; HDAC8; HDAC9 \| \| AS8351 \| 4.63 \| histone demethylase \| \| SGI-1776 free base \| 4.63 \| FLT3; Pim1; Pim2; Pim3 \| \| CUDC101 \| 4.63 \| EGFR; HDAC; HDAC1; HDAC10; HDAC2; HDAC3; HDAC4; HDAC5; HDAC6; HDAC7; HDAC8; HDAC9; HER2 \| \| LMK235 \| 4.62 \| HDAC4; HDAC5 \| \| Seclidemstat \| 4.62 \| LSD1 \| \| ENMD2076 \| 4.59 \| Aurora A; RET; FLT3; Src; VEGFR3/FLT4 \| \| I-BET151 \| 4.58 \| BRD2; BRD3; BRD4 \| \| (S)-(+)-Camptothecin \| 4.57 \| Topo I \| \| MK8745 \| 4.57 \| Aurora A; Aurora B \| \| Topotecan hydrochloride \| 4.56 \| Topo I (DU-145 Luc cells); Topo I (MCF-7 Luc cells) \| \| PI3K/HDAC Inhibitor \| 4.56 \| HDAC1; HDAC10; HDAC11; HDAC2; HDAC3 \| \| 5-Fluorouridine \| 4.55 \| DNA synthesis; Upp \| \| Fluorouracil \| 4.53 \| Thymidylate synthase \| \| Minomustine \| 4.51 \| HDAC1; HDAC2; HDAC3; HDAC6; HDAC8 \| \| Daunorubicin hydrochloride \| 4.50 \| DNA synthesis \| \| Cerdulatinib \| 4.50 \| JAK1; JAKs; Syk \| \| Salermide \| 4.49 \| SIRT1; SIRT2 \| \| BG45 \| 4.47 \| HDAC1; HDAC2; HDAC3; HDAC6 \| \| Rucaparib Phosphate \| 4.46 \| PARP \| \| Chelerythrine chloride \| 4.44 \| PKC \| \| Podophyllotoxin \| 4.44 \| microtubule; Topo II \| \| JIB04 \| 4.44 \| JMJD2A; JMJD2B; JMJD2D; JMJD2E; JARID1A \| \| Panobinostat \| 4.43 \| HDAC (MOLT-4 cells); HDAC (Reh cells) \| \| Talazoparib \| 4.35 \| PARP \| \| UNC0638 \| 4.34 \| G9a; GLP \| \| Tubastatin A HCl \| 4.33 \| HDAC1; HDAC2; HDAC3; HDAC6; HDAC8 \| \| Irinotecan hydrochloride trihydrate \| 4.32 \| Topo I \| \| Romidepsin \| 4.31 \| HDAC1; HDAC2 \| \| KW2449 \| 4.30 \| Aurora A; Abl; Abl (T315I); Kit; FGFR1; FLT3; FLT3 (D835Y); JAK2; Src \| \| Danusertib \| 4.28 \| Aurora A; Aurora B; Aurora C; Abl; RET; FGFR1; TrkA \| \| AT9283 \| 4.26 \| Aurora A; Aurora B; Abl1 (T315I); JAK2; JAK3 \| \| Cytarabine \| 4.24 \| DNA synthesis \| \| ICG001 \| 4.20 \| Wnt/β-catenin;CBP \| \| HTH-01-015 \| 4.19 \| NUAK1; NUAK2 \| \| (+)-JQ-1 \| 4.18 \| BRD4 (1); BRD4 (2) \| \| Epirubicin hydrochloride \| 4.12 \| Topo \| \| Sodium Aescinate \| 4.12 \| HIF-1α \| \| C660-0753 \| 4.10 \| Ataxin-2 \| \| AZD1208 \| 4.09 \| Pim1; Pim2; Pim3 \| \| dBET6 \| 4.09 \| BET \| \| Citarinostat \| 4.08 \| HDAC1; HDAC2; HDAC3; HDAC6; HDAC8 \| \| RGB-286638 free base \| 4.07 \| CDK1/cyclinB1; CDK2/cyclinE; CDK3/cyclinE; CDK4/cyclinD1; CDK9/cyclinT1; p35-CDK5 \| \| ARV-825 \| 4.06 \| BRD4 \| \| D717-0088 \| 4.05 \| Ataxin-2 \| \| Cucurbitacin B \| 4.02 \| HIF-1; PTEN; STAT3 \| |  |  |

**Supplementary Table 2**. Primers for real-time polymerase chain reaction analysis

| Gene | species | | Forward (5’ to 3’) | Reverse (5’ to 3’) |
| --- | --- | --- | --- | --- |
| *Homx1* | Rattus | AGGTGCACATCCGTGCAGAG | | CTTCCAGGGCCGTATAGATATGGTA |
| *Ptgs2* | Rattus | CTCAGCCATGCAGCAAATCC | | GGGTGGGCTTCAGCAGTAAT |
| *Nampt* | Rattus | CCCAATTGAAGTAAAGGCTGT | | TGGTGAGCCAGTAGCACTCTG |
| *Naprt* | Rattus | CGACCTCTACCAGGCTACAAT | | CAAAGGAACCTCCGAAGGGG |
| *Sirt1* | Rattus | TAGCTTTGTCAGATAAGGAAGGA | | ACAGCTTCACAATCAACTTTGT |
| *Gapdh* | Rattus | AGGTCGGTGTGAACGGATTTG | | TGTAGACCATGTAGTTGAGGTCA |
| *Ptgs2* | Mouse | TGAGCAACTATTCCAAACCAGC | | GCACGTAGTCTTCGATCACTATC |
| *Homx1* | Mouse | AAGCCGAGAATGCTGAGTTCA | | GCCGTGTAGATATGGTACAAGGA |
| *Nppa* | Mouse | TCGTCTTGGCCTTTTGGCT | | TCCAGGTGGTCTAGCAGGTTCT |
| *Nppb* | Mouse | GAGGTCACTCCTATCCTCTGG | | GCCATTTCCTCCGACTTTTCTC |
| *Myh7* | Mouse | ACTGTCAACACTAAGAGGGTCA | | TTGGATGATTTGATCTTCCAGGG |
| *Nampt* | Mouse | CAGAAGCCGAGTTCAACATC | | CGGCATTCAAAGTAGGAATAAAC |
| *Naprt* | Mouse | AGCCTCGTAGCCACCAATG | | CCGCAAACCCATCTCTAGTAGT |
| *Sirt1* | Mouse | TAGCTTTGTCAGATAAGGAAGGA | | ACAGCTTCACAATCAACTTTGT |
| *Gapdh* | Mouse | AGGTCGGTGTGAACGGATTTG | | TGTAGACCATGTAGTTGAGGTCA |

**Supplementary Table 3**. Primers for ChIP-PCR

| Gene | species | | Forward (5’ to 3’) | Reverse (5’ to 3’) |
| --- | --- | --- | --- | --- |
| *NAPRT* | Rattus | ATGTGCAGTTCCTGGCTTCAG | | CGAGGGAACCCTCCGGCAGG |
