# Supplementary material for: (+)-JQ-1 alleviates cardiac injury in myocardial infarction by inhibiting ferroptosis through the NAMPT/SIRT1 pathway
Source: Cell Death Dis. 2025 Jul 23;16(1):548. doi: 10.1038/s41419-025-07880-x (PMC12283960; doi:10.1038/s41419-025-07880-x)

Figure 1F

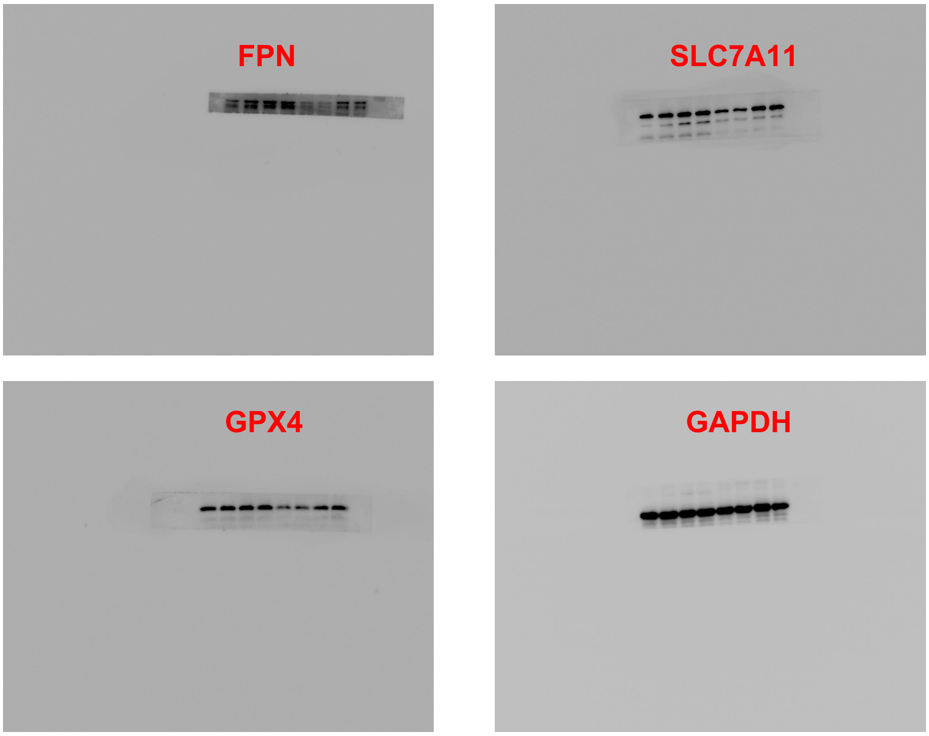

Figure 3M

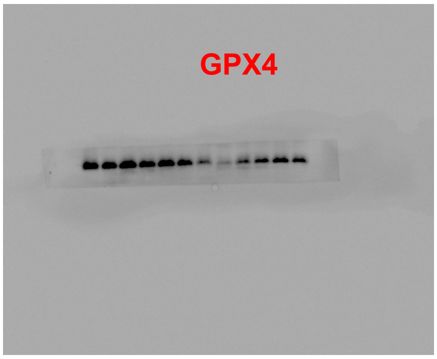

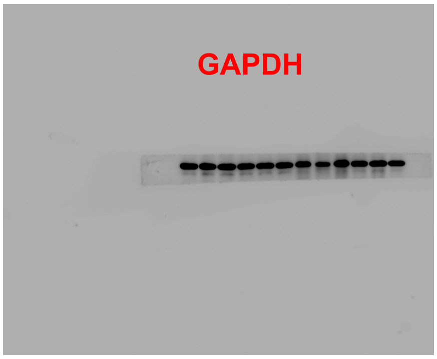

Figure 5H

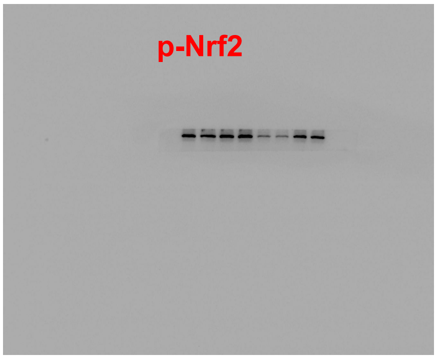

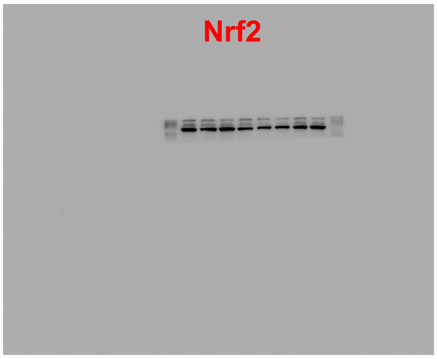

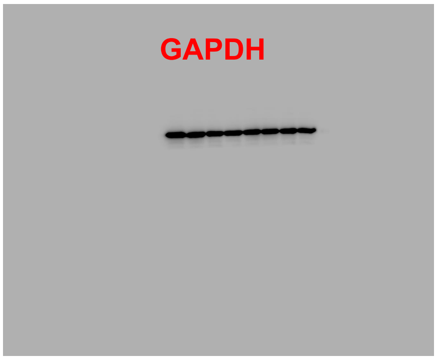

Figure 5J

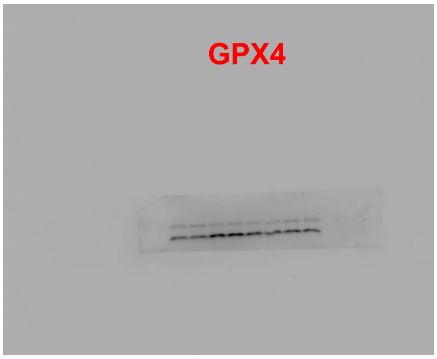

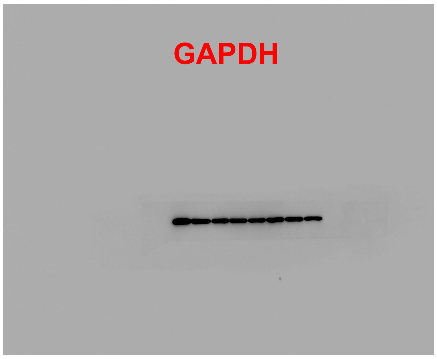

Figure 6H

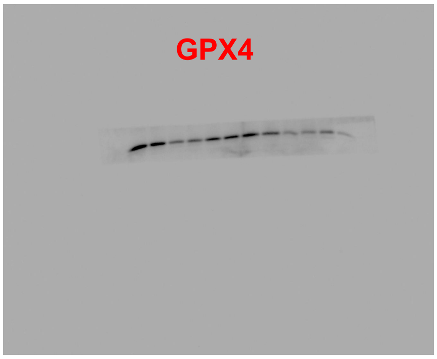

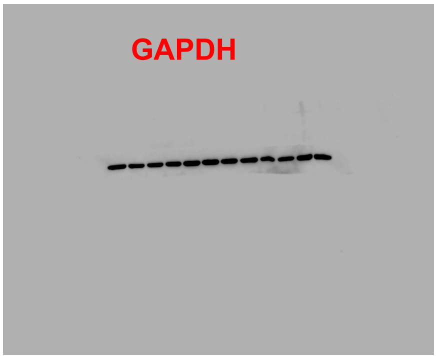

Figure 7J

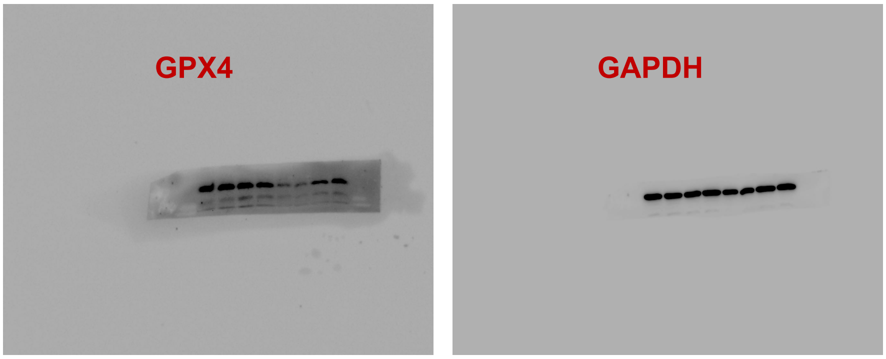

Supplemental Figure.5F

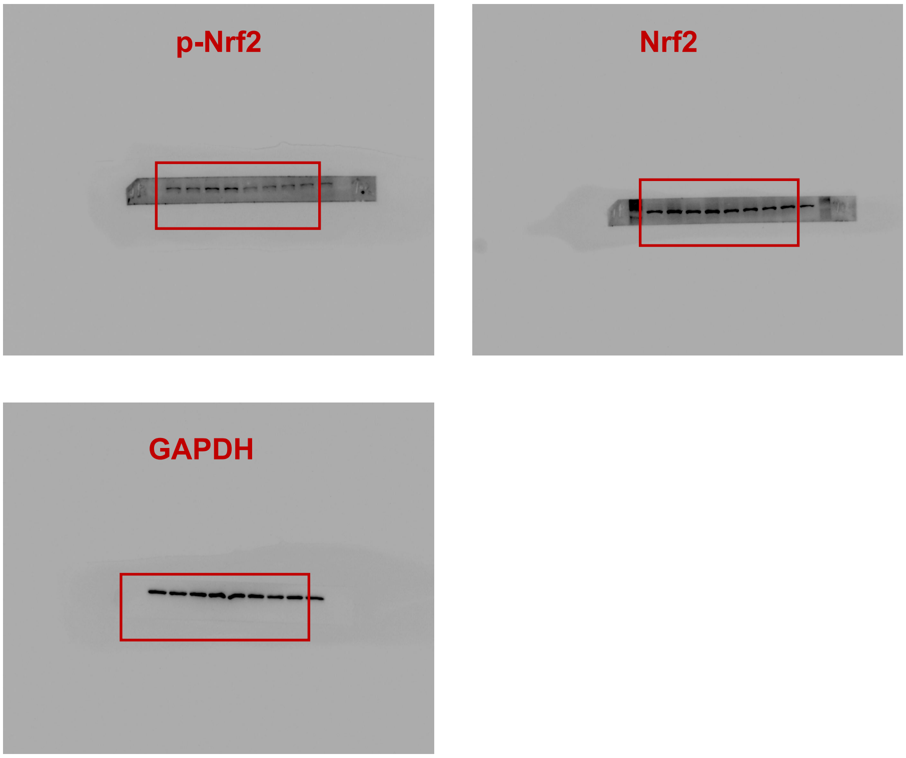

Supplemental Figure.6A

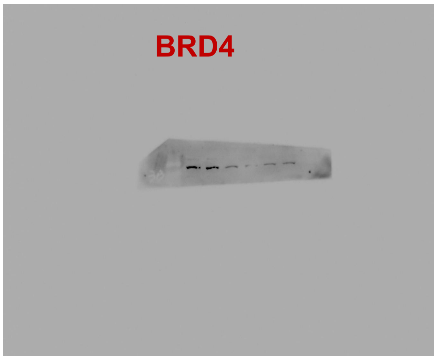

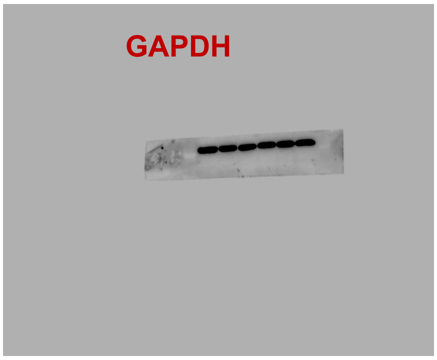

Supplement: Supplementary file 2 — uncropped original western blots [file 41419_2025_7880_MOESM2_ESM.pdf]
